# PP4 Is Essential for Germinal Center Formation and Class Switch Recombination in Mice

**DOI:** 10.1371/journal.pone.0107505

**Published:** 2014-09-12

**Authors:** Ming-Yu Chen, Ya-Ping Chen, Ming-Sian Wu, Guanni-Yi Yu, Wen-Jye Lin, Tse-Hua Tan, Yu-Wen Su

**Affiliations:** 1 Immunology Research Center, National Health Research Institutes, Zhunan, Miaoli County, Taiwan; 2 National Institute of Infectious Diseases and Vaccinology, National Health Research Institutes, Zhunan, Miaoli County, Taiwan; 3 Department of Pathology & Immunology, Baylor College of Medicine, Houston, Texas, United States of America; Institut de Recherches Cliniques de Montréal (IRCM), Canada

## Abstract

PP4 is a serine/threonine phosphatase required for immunoglobulin (Ig) VDJ recombination and pro-B/pre-B cell development in mice. To elucidate the role of PP4 in mature B cells, we ablated the catalytic subunit of murine PP4 *in* *vivo* utilizing the CD23 promoter and cre-loxP recombination and generated CD23^cre^PP4^F/F^ mice. The development of follicular and marginal zone B cells was unaffected in these mutants, but the proliferation of mature PP4-deficient B cells stimulated by *in* *vitro* treatment with either anti-IgM antibody (Ab) or LPS was partially impaired. Interestingly, the induction of CD80 and CD86 expression on these stimulated B cells was normal. Basal levels of serum Igs of all isotypes were strongly reduced in CD23^cre^PP4^F/F^ mice, and their B cells showed a reduced efficiency of class switch recombination (CSR) *in* *vitro* upon stimulation by LPS or LPS plus IL-4. When CD23^cre^PP4^F/F^ mice were challenged with either the T cell-dependent antigen TNP-KLH or the T cell-independent antigen TNP-Ficoll, or by H1N1 virus infection, the mutant animals failed to form germinal centers (GCs) in the spleen and the draining mediastinal lymph nodes, and did not efficiently mount antigen-specific humoral responses. In the resting state, PP4-deficient B cells exhibited pre-existing DNA fragmentation. Upon stimulation by DNA-damaging drug etoposide *in* *vitro*, mutant B cells showed increased cleavage of caspase 3. In addition, the mutant B cells displayed impaired CD40-mediated MAPK activation, abnormal IgM-mediated NF-κB activation, and reduced S phase entry upon IgM/CD40-stimulation. Taken together, our results establish a novel role for PP4 in CSR, and reveal crucial functions for PP4 in the maintenance of genomic stability, GC formation, and B cell-mediated immune responses.

## Introduction

Protein phosphatase 4 (PP4) is a complex and well-conserved holoenzyme containing a catalytic subunit that can bind to diverse regulatory subunits depending on the biological circumstances. The catalytic subunit of PP4, termed PP4c, belongs to the type 2A serine/threonine protein phosphatase (PP2A) family. In *C. elegans*, the PP4 holoenzyme is required for centrosome maturation during mitosis and sperm meiosis [1]. In mammals, PP4 participates in a number of processes essential for normal cellular physiology, including microtubule organization [2], [3], homologous recombination (HR)-mediated DNA repair [4], [5], the DNA damage response [6]–[10], histone modification [11], apoptosis [12], [13], pre-TCR signaling [14], TNF signaling [15], [16], Toll-like receptor (TLR)-4 signaling [17], and NF-κB regulation [18]–[20]. Germline deletion of PP4 in mice is embryonic lethal [14], and conditional deletion of PP4 specifically in murine T cells severely impairs T cell development [14]. We previously ablated PP4 specifically in developing B cells using the mb-1/cre/loxP system and generated mb-1/cre/PP4^F/F^ mice [21]. We found that this loss of PP4 impaired immunoglobulin (Ig) VDJ recombination and led to a profound block in B cell development at the pro-B/pre-B cell stage [21]. In pro-B cells of these mutants, DNA breaks in the Ig loci were drastically increased, suggesting a role for PP4 in non-homologous end joining (NHEJ) DNA repair. NHEJ repair is the major repair pathway, which implicates in class switching [22]. Our earlier finding raises the question whether PP4 deficiency affects Ig class switch recombination. However, because mb-1/cre/PP4^F/F^ mice do not produce mature B cells, they cannot be used to examine the role of PP4 in events triggered by antigen encounter.

After immunization or infection, activated B cells migrate to the T cell border in a primary follicle within a secondary lymphoid organ searching for T cell help [23], [24]. The interaction between CD40 ligand (CD40L) on follicular helper T (T_FH_) cells and CD40 on these activated B cells is essential for antigen-specific proliferation and germinal center (GC) formation [25]. Activated B cells also undergo Ig class switch recombination (CSR) in which the gene segment encoding the original constant (C) µ region of the Ig is excised from the DNA and replaced with one of the available 3′ gene segments: Cγ, Cε or Cα. The progeny of the original IgM-expressing B cells thus express IgG, IgE or IgA instead of IgM [26]. This class switching is critical for matching antibody (Ab) effector functions to the type of antigen encountered during an immune response. In addition, activated B cells rapidly upregulate the costimulatory molecules B7-1/CD80 and B7-2/CD86, which are also required for the GC reaction because they promote the antigen-presenting capacity of the B cells and support a bi-directional interaction between these B cells and T_FH_ cells [27]–[29]. B cells selected by T_FH_ cells undergo somatic hypermutation (SHM) during the GC reaction. Via SHM, B cells expressing B cell receptors (BCRs) of high affinity for the cognate antigen gain a survival advantage and mount a primary humoral response. Affinity-matured class-switched B cells then exit the GC and differentiate into either plasma cells or memory B cells, enabling efficient and effective Ab protection both immediately and in the future [30], [31].

To investigate the role of PP4 in mature B cells, we examined conditional knockout mice (CD23^cre^PP4^F/F^ mice) created by crossing B-cell specific CD23^cre^ mice to mice bearing a floxed PP4 gene (PP4^F/F^ mice) [14], [32]. Although B cell maturation into follicular (FO) and marginal zone (MZ) B cells was unaffected in CD23^cre^PP4^F/F^ mice, peripheral B cells of these mutants showed defects in CSR, the GC reaction, and DNA repair. As a result, humoral responses were compromised in CD23^cre^PP4^F/F^ mice. Our data reveal PP4 as a crucial player in the maintenance of genomic stability, GC B cell differentiation, Ig CSR, and B cell-mediated immune responses.

## Methods

### Mice

PP4^F/F^ mice [14], CD23^cre^ mice [32], and BCR^HEL^ transgenic mice [33] generated as previously described were maintained in the Laboratory Animal Center of the National Health Research Institute (NHRI). The CD23^cre^ mice and BCR^HEL^ transgenic mice used in all experiments were heterozygous for the transgenes. 8–12 weeks old, age-matched mice were used in all experiments. All mice were euthanized by carbon dioxide inhalation. Mice number in each experiment was specified in the Figure Legends. Virus infection in mice was performed in strict accordance with the recommendations in the Regulations for Using the Infectious Animal Room of NHRI Laboratory Animal Center. Infectious animal experiment area, which is compliance with the standards of level P2 as a laboratory for infectious elements, is monitored every three months. All animal studies were reviewed and approved by NHRI’s Institutional Animal Care and Use Committee (Permit Number: 099111-A, 102006 and 103086-A), and all efforts were made to minimize mice suffering.

### B cell purification and cell culture

Single cell suspensions were prepared from the spleens of mice by sieving and gentle pipetting through 70-µm nylon mesh filters (Falcon; BD Biosciences). Non-B cells were depleted using a cocktail of biotin-conjugated Abs recognizing NK1.1 (PK136), Thy1.2 (30-H12), Gr1 (RB6-8C5), CD11b (M1/70), CD11c (HL3) or Ter-119 (BD Biosciences), followed by application of IMag Streptavidin Particles Plus beads (BD Biosciences). Purified splenic B cells were cultured in RPMI 1640 medium supplemented with 2 mM L-glutamine (Invitrogen), 100 U penicillin-streptomycin (Invitrogen), 100 mM Hepes (Sigma-Aldrich), 0.055 mM 2-mercaptoethanol (Gibco), and 10% FBS (Hyclone). Splenic B cells of purity between 94–98% were used in all experiments.

### BrdU incorporation

For *in* *vivo* BrdU incorporation, mice were injected intraperitoneally (i.p.) with 2 mg BrdU (Sigma) in 200 µl sterile PBS twice daily (8 h apart) for 3 or 4 consecutive days. Splenic B cells were sorted by FACSAria II (BD). For *in* *vitro* BrdU incorporation, splenic B cells were cultured in RPMI medium supplemented with 10 µM BrdU for 2 or 3 days. In both cases, cells were analyzed using a BrdU-Flow kit (BD Pharmingen).

### Induction of Ig class switching

Purified splenic B cells were seeded at a density of 1×10^6^/ml and cultured for 4 days in RPMI medium containing either 50 µg/ml lipopolysaccharide (LPS; InvivoGen) to induce switching from IgM to IgG_3_, or in RPMI medium containing 50 µg/ml LPS plus 10 ng/ml IL-4 (PeproTech) to induce switching from IgM to IgG_1_ [34]. Cells exhibiting switching and thus producing new Ig classes were identified by FACS analysis as described below.

### Digestion-circularization (DC)-PCR

Class switching in B cells treated as described above was confirmed by DC-PCR. To detect the switched Sµ-Sγ3 sequence (see Results section) by DC-PCR, genomic DNA extracted from B cells was digested with *Eco*RI, and self-ligated to form a circular DNA that acted as template for nested PCR, as previously described [35]. To detect the switched Sµ-Sγ1 sequence, genomic DNA extracted from B cells stimulated with 50 µg/ml LPS plus 10 ng/ml IL-4 for 4 days were treated using a protocol modified from a previous report [36] and illustrated in Figure S1. DC-PCR of the nicotinic acetylcholine receptor beta subunit gene (nAChRe) was performed as a loading control, as previously described [37].

### DNA damage detection by comet assay

To perform comet assays, genomic DNA from splenic B cells was processed using the OxiSelect Comet Assay Kit following the manufacturer’s instructions (Cell Biolabs). Data were analyzed using Comet Assay IV v4.3 software (PERCEPTIVE Ins.).

### Flow cytometric analyses (FACS)

Single cell suspensions of 1×10^6^ cells were washed twice with FACS buffer (2% BSA/2 mM EDTA/PBS, 0.1% NaN_3_) and maintained in the dark at 4°C throughout experiments. Flow cytometric data were acquired using a CantoII flow cytometer and FACSDiva software (both from BD Biosciences). FlowJo software (Tree Star, Inc.) was used for data analyses.

For marker expression determinations, cells were incubated for 15 min on ice with anti-mouse Abs, including IgG_1_ (RMG1-1)-FITC, IgD (11-26c.2a)-Pacific Blue (both from Biolegend), CD38 (90)-PE, CD86 (GL1)-eFluor 450, CXCR4 (2B11)-PE, IgM (eB121-15F9)-eFluor 450 (all from eBioscience), B220 (RA3-6B2)-APC/APC-Cy7, CD21 (7G6)-PE, CD23 (B3B4)-PE-Cy7, CD25 (PC61)-APC-Cy7, CD40 (3/23)-FITC, CD95 (Jo2)-PE-Cy7, GL7-FITC, IgD (11-26c.2a)-FITC/PE, IgM (II/41)-FITC/APC/PE-Cy7, IgG_3_ (R40-82)-biotin, streptavidin-APC (all from BD Biosciences), and PNA-FITC (Vector). Immunostained cells were washed twice in FACS buffer prior to incubation with 7AAD (Sigma). Viable cells were gated from the 7AAD-negative population prior to analysis.

For intracellular staining to detect caspase 3 cleavage, cells were fixed and permeabilized using the BrdU-Flow kit (BD Pharmingen) prior to incubation with PE-conjugated Ab specifically recognizing cleaved caspase 3 (BD Biosciences).

### Immunohistochemistry (IHC)

Mouse spleens were fixed, embedded in paraffin, and sectioned with a microtome set to 5 µm. After deparaffinization and rehydration, spleen sections were retrieved by 10 mM sodium citrate, and incubated in Protein Block Serum-Free (Dako). For primary Ab binding, blocked slides were incubated overnight at 4°C with rat anti-mouse B220 (BD Biosciences) and PNA-biotin (Vector Laboratories). For secondary Ab binding, slides were incubated at RT for 60 min with biotin-conjugated donkey anti-rat IgG (Research Diagnostics) and alkaline phosphatase (AP)-conjugated streptavidin (Molecular Probes). Biotin-conjugated signals were detected with VECTASTAIN Elite ABC reagent (Vector Laboratories) and DAB substrate (Vector Laboratories) according to the manufacturer’s instructions. AP-conjugated signals were visualized by incubating slides at RT for 30 min with NBT/BCIP solution (Roche). Sections were dehydrated, mounted using IMMU-MOUNT (Thermo Fisher Scientific), and scanned by the image capture device (Leica SCN 400) of 40x magnification.

### Immune responses to antigens or H1N1 virus infection

To examine immune responses to the T-independent (Ti) antigen 2,4,6-trinitrophenyl (TNP)-Ficoll, mice were i.p. immunized with 0.5 µg/g body weight TNP-Ficoll (BIOSEARCH). Serum samples at day 0 (pre-immunization) and days 7, 14, 21 and 28 post-immunization were analyzed by standard ELISA for determination of TNP-specific Igs. In brief, TNP-BSA (BIOSEARCH) in carbonate buffer (Sigma) was coated in 96-well plate and blocked with 3% BSA. Serum samples which were serially diluted in 1% BSA/PBS were added to plate and incubated overnight. After washing steps, goat anti-mouse Ig isotype specific antibodies (Southern Biotech) were added to wells to detect bound immunoglobulin. ELISAs were developed using TMB Substrate Reagent Set (BD) and read at 450 nm. To examine immune responses to the T-dependent (Td) antigen TNP-Keyhole Limpet Hemocyanin (TNP-KLH, BIOSEARCH), mice were i.p. immunized with 5 µg/g body weight TNP-KLH in alum (Sigma). Serum samples at day 0 (pre-immunization) and days 7, 14, 21, 35 and 49 post-immunization were analyzed by ELISA as above. Spleen samples were harvested at days 0 and 14 post-immunization for FACS and IHC analyses as described above. To examine immune responses to hen egg lysozyme (HEL), mice were i.p. immunized with 70 µg/mouse HEL (Sigma) in alum. Spleen samples were harvested at days 0 and 7 post-immunization for FACS analyses as described above.

To examine immune responses to H1N1 virus infection, mice were anesthetized by 1–5% isofluorane (Aesica Queenborough, UK) and infected intranasally with a sublethal dose (5×10^3^ pfu/mouse) of the influenza virus, strain A/PR8/34/H1N1 (obtained from Dr. Shin-Ru Shih), which was amplified in MDCK cells. The virus titer was determined by plaque assay as described previously [38]. The body weight of infected animals was monitored daily. Mice that lost ≥30% of their original body weight and/or displayed symptoms of inactivity, labored respiration and huddling behavior were euthanized. Experiments involving influenza were terminated before or at day 15 after infection. To determine H1N1-specific Igs level, serum samples at days 0 and 9 post-infection were collected and analyzed by standard ELISA on 96-well plates coated with A/PR8/34/H1N1 Hemagglutinin (HA) protein (Sino Biological Inc.).

### RT-PCR

To examine levels of germline and class-switched Ig transcripts, PCR oligomer primers and reactions conditions were employed as previously reported [34]. PCR reactions were performed using mRNA extracted from B cells that had been left unstimulated, or stimulated for 4 days with LPS alone or with LPS plus IL-4 as described above.

### Western blotting

Splenic B cells (10^7^) were left unstimulated, or pre-incubated on ice for 30 min with 10 µg/ml anti-mouse IgM-biotin (Southern Biotech) or anti-mouse CD40-biotin (3/23; BD Biosciences), followed by crosslinking with 1 µg/ml streptavidin (Sigma). Cells were lysed in 100 µl 1% NP-40 lysis buffer [10 mM Hepes, 10 mM KCl, 0.1 mM EDTA, 0.1 mM EGTA, with protease inhibitor freshly added]. Lysates were then fractionated by SDS-PAGE and subjected to Western blotting using standard procedures. Proteins were detected using anti-phospho-Erk1/2 (T202/Y204), anti-Erk1/2, anti-phospho-JNK (T183/Y185), anti-JNK, anti-phospho-Akt/PKB (S473), anti-Akt/PKB, anti-IκB (all from Cell Signaling), or anti-gp-96 (Invitrogen) Abs. Blots were developed using ECL Plus Western Blotting Detection Reagents (Amersham).

### Statistical analysis

Data were analyzed using a one-tailed distribution, type 3 Student’s *t*-test. Differences between treatment groups with *p*-values≤0.05 were considered statistically significant. Symbols of *p*-values were defined as: *, *p*≤0.05; **, *p*≤0.005 and ***, *p*≤0.0005.

## Results

### PP4 deficiency does not affect B cell maturation but impairs BCR-mediated proliferation

**Figure 1:**
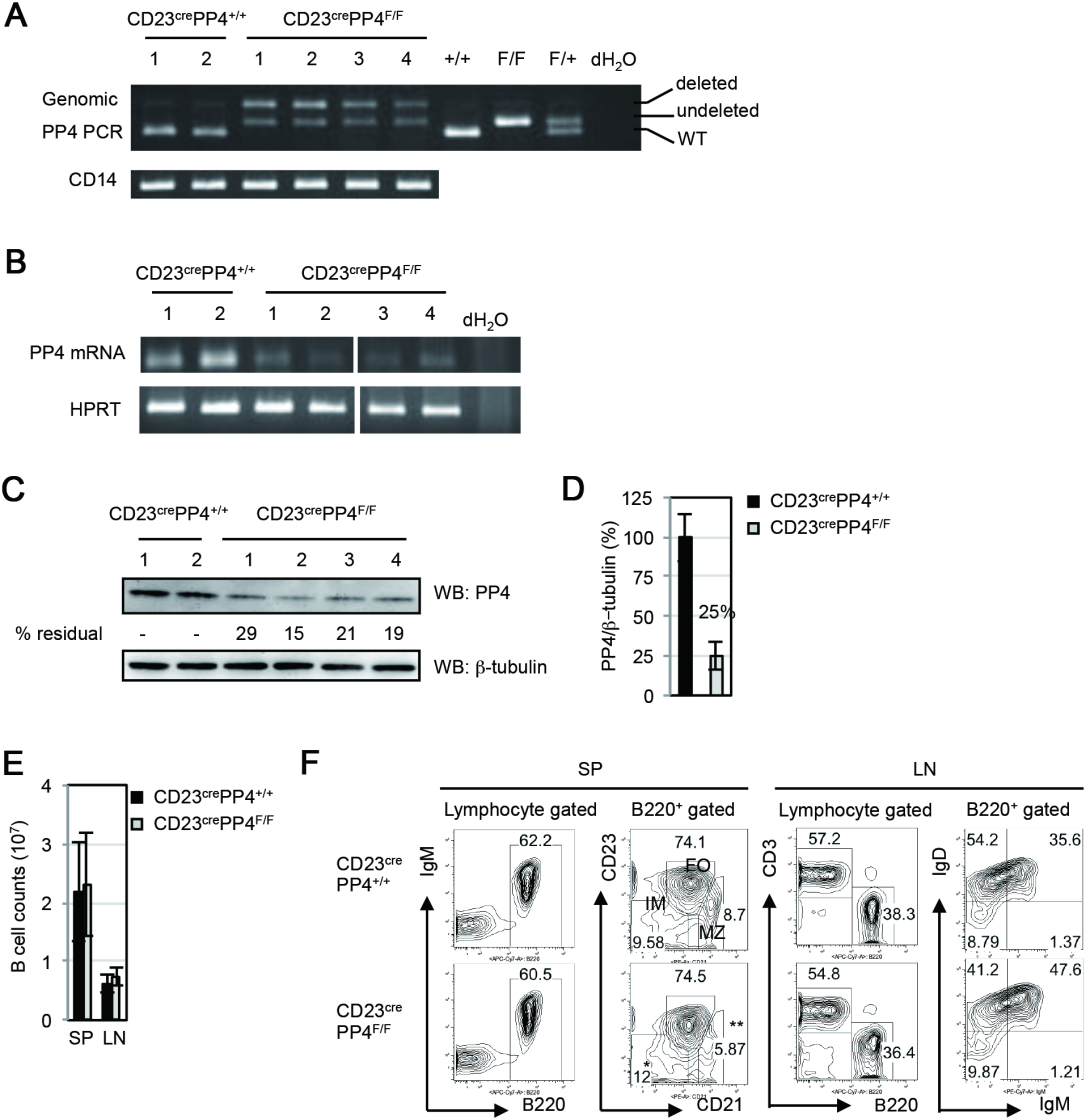
Efficiency of *pp4c* deletion. (**A**) Top panel: PCR analysis of genomic DNA from splenic B cells of WT (n = 2) and CD23^cre^PP4^F/F^ (n = 4) mice showing products representing the WT *pp4c* allele (+/+), the *pp4c* floxp allele (undeleted F/F), and the deleted *pp4c* allele (deleted F/F). Numbers above the top panel are individual mouse labels. Bottom panel: PCR analysis of CD14 as a loading control. (**B**) Top panel: RT-PCR analysis of mRNA from splenic B cells from the WT and CD23^cre^PP4^F/F^ mice in (A). Bottom panel: HPRT, loading control. (**C**) Top panel: Western blot to detect PP4 protein in the spenic B cells in (B). Numbers below this panel (% residual) are the relative protein levels quantified by Image J and normalized to the WT value (set to 100%). Bottom panel: β-tubulin, loading control. (**D**) Quantitation of the PP4 protein levels in the B cells in (C) after normalization to β-tubulin values. Data are the mean ± SD of samples assayed per group. For (A–D), results are representative of two independent experiments. (**E**) Quantitation of peripheral B cell counts in the spleen (SP) and lymph nodes (LN) of WT and CD23^cre^PP4^F/F^ mice (n = 40–50 mice/group). Data are the mean ± SD. (**F**) FACS profiles of B220 vs IgM and CD21 vs CD23 expression by WT and CD23^cre^PP4^F/F^ splenocytes (SP), and of B220 vs CD3 and IgM vs IgD expression by WT and CD23^cre^PP4^F/F^ total mesenteric LN cells. Numbers in quadrants represent the percentage of the indicated population relative to the total. Results are representative of 3 mice/group and of three independent experiments.

To study the role of PP4 in peripheral B cells *in* *vivo*, we bred CD23^cre^ knock-in mice [32] with *pp4 loxp*-flanked (*floxp*) mice [14] to generate CD23^cre^PP4^F/F^ mice (Figure S2A). Littermate of CD23^cre^ mice carrying *pp4* wild type (designated as CD23^cre^PP4^+/+^ or WT mice) were utilized as controls in all experiments. Immature (IM) B cells in the spleen differentiate into follicular (FO) B cells, characterized by the B220^+^CD21^int^CD23^high^ population, and marginal zone (MZ) B cells, as B220^+^CD21^high^CD23^low^ subset, respectively, which govern a protective innate and adaptive immune response upon antigen encounter [39]. It has previously been demonstrated that, in the spleen, CD23-controlled cre activity initiates in the IgM^+^IgD^low^ immature (IM) B cell population and increases in IgM^low^IgD^high^ mature B cells [32]. We found, using genomic PCR analysis, that *pp4 floxp* in B cells of CD23^cre^PP4^F/F^ mice was partially deleted (Figure 1A), leading to a strong reduction of PP4 mRNA in CD23^cre^PP4^F/F^ B cells (Figure 1B). To determine PP4 protein levels in CD23^cre^PP4^F/F^ B cells, we employed an in-house rabbit polyclonal anti-PP4 Ab that binds to a PP4-Flag fusion protein of 34 kDa in 293T cells (Figure S2B). This Ab does not recognize the catalytic subunit of PP2A (PP2Ac), which is closely related to PP4c but has a molecular weight of 38 kDa (Figure S2C). Western blotting using this anti-PP4 Ab showed that PP4 protein expression in CD23^cre^PP4^F/F^ B cells reached only about 25% of WT levels (Figure 1C, 1D). These data indicate that our strategy to eliminate PP4 expression in peripheral B cells was largely successful.

CD23^cre^PP4^F/F^ mice were viable and fertile, and total B cell counts in the spleen and lymph nodes (LN) of these mutants were similar to those in WT mice (Figure 1E). The B220^+^ compartment comprised 60.5% of total splenocytes in CD23^cre^PP4^F/F^ mice, a proportion similar to that in WT mice (Figure 1F). We detected a minor accumulation of B220^+^CD23^−^CD21^−^ IM B cells and a slight reduction in B220^+^CD23^low^CD21^high^ MZ B cells in spleens of CD23^cre^PP4^F/F^ mice but these differences were not statistically significant (Figure 1F). The generation of B220^+^CD23^high^CD21^low^ FO B cells was also comparable in WT and CD23^cre^PP4^F/F^ spleens. In LN, the FACS profiles of B220 versus CD3 expression by total lymphocytes, as well as IgM versus IgD expression by total B cells, were comparable to those in WT mice (Figure 1F). Thus, loss of PP4 does not impair B cell maturation or affect steady-state peripheral B cell populations.

**Figure 2:**
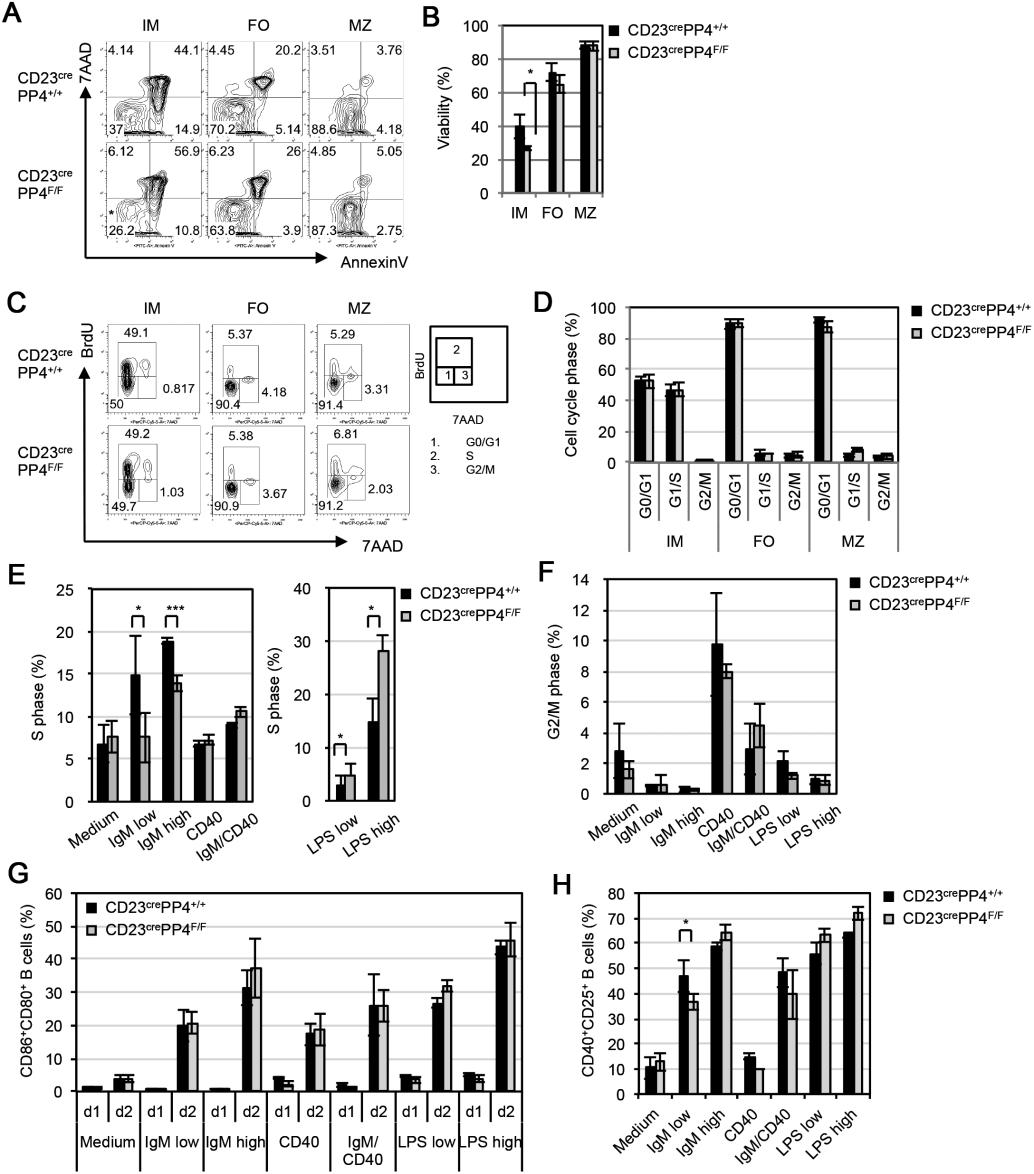
Characterization of B cells in CD23^cre^PP4^F/F^ mice. (**A**) FACS assay of apoptosis of splenic B cells that were freshly isolated from WT and CD23^cre^PP4^F/F^ mice (n = 3/group) and subjected to AnnexinV vs 7AAD staining. Viabilities are shown for the immature (IM), follicular (FO) and marginal zone (MZ) B cell populations. Numbers in quadrants represent the percentage of the indicated population relative to the total. (**B**) Quantitation of the percentage of viable (AnnexinV^−^7AAD^−^) cells in the IM, FO and MZ B cell populations in (A). Data are the mean ± SD of triplicate samples from each group. For (A–B), results are representative of two independent experiments. (**C**) *In* *vivo* BrdU incorporation assay. Left panel: WT and CD23^cre^PP4^F/F^ mice (n = 3/group) were i.p. injected with BrdU twice daily (8 h apart) on 3 consecutive days. IM, FO and MZ splenocytes were freshly sorted, subjected to intracellular staining with anti-BrdU plus 7AAD, and analyzed by FACS. Right panel: Diagram indicating relative position of specific mitotic phase cells in the FACS profiles in the left panel. (**D**) Quantitation of the results in (C) showing the percentages of cells in the G0/G1, S and G2/M phases. Data are the mean ± SD of triplicate samples from each group. For (C–D), results are representative of two independent experiments. (**E**) *In* *vitro* BrdU incorporation assay. Splenic B cells were freshly isolated from WT (n = 3) or CD23^cre^PP4^F/F^ (n = 4) mice and cultured in BrdU-containing maintenance medium alone (Medium), or in maintenance medium containing 1 µg/ml anti-IgM (IgM low), 10 µg/ml anti-IgM (IgM high), 5 µg/ml anti-CD40 Ab (CD40), 1 µg/ml anti-IgM plus anti-CD40 Abs (IgM/CD40), 1 µg/ml LPS (LPS low), or 10 µg/ml LPS (LPS high). After 48 h, cells were analyzed by FACS. Data shown are the quantitation of the mean percentage ± SD of B cells in S phase relative to total B cells after 48 h stimulation. (**F**) Quantitation of the mean percentage ± SD of B cells in G2/M phase from the data in (E) after 48 h stimulation. (**G**) Quantitation of the mean percentage ± SD of activated (CD80^+^CD86^+^) B cells from the data in (E) after 24 h (d1) or 48 h (d2) of stimulation. (**H**) Quantitation of the mean percentage ± SD of CD40^+^CD25^+^ B cells from the data in (G) after 48 h (d2) stimulation. For (E–H), results are representative of two independent experiments.

To determine whether PP4 deficiency disrupted B cell homeostasis *in* *vivo*, we isolated IM, FO and MZ B cells from spleens of WT and CD23^cre^PP4^F/F^ mice and subjected them to apoptosis assays and BrdU incorporation analysis. The proportions of viable (AnnexinV^−^7AAD^−^) B cells recovered from the mutant mice were comparable to those in WT mice (Figure 2A, 2B). While a slight reduction in the viability of mutant IM B cells was observed (Figure 2A, 2B), no cell cycle arrest was observed in CD23^cre^PP4^F/F^ IM, FO or MZ B cells (Figure 2C, 2D). To examine whether PP4 deficiency affected B cell proliferation *in* *vitro*, WT and CD23^cre^PP4^F/F^ B cells were left unstimulated, or stimulated with anti-IgM, anti-CD40, anti-IgM plus anti-CD40, or LPS for 48 h in full medium supplemented with BrdU. PP4-deficient B cells showed reduced S phase entry upon IgM stimulation but increased S phase entry upon LPS stimulation (Figure 2E). No cell cycle arrest in the G2/M phase was observed in the mutant B cells (Figure 2F). Interestingly, similar surface levels of the B cell activation markers CD80 and CD86 were induced on mutant and WT B cells exposed to the above stimuli (Figure 2G, 2H). Although a mild reduction in the CD40^+^CD25^+^ population of activated B cells was found in mutant cultures treated with a low dose of anti-IgM (1 µg/ml), this difference was not apparent upon stimulation with a high anti-IgM dose (10 µg/ml) (Figure 2H and Figure S3A). These results demonstrate that PP4 deficiency does not impair B cell maturation but does alter cell cycle entry in response to stimulation with either anti-IgM or LPS.

### CSR efficiency is reduced in PP4-deficient B cells

**Figure 3:**
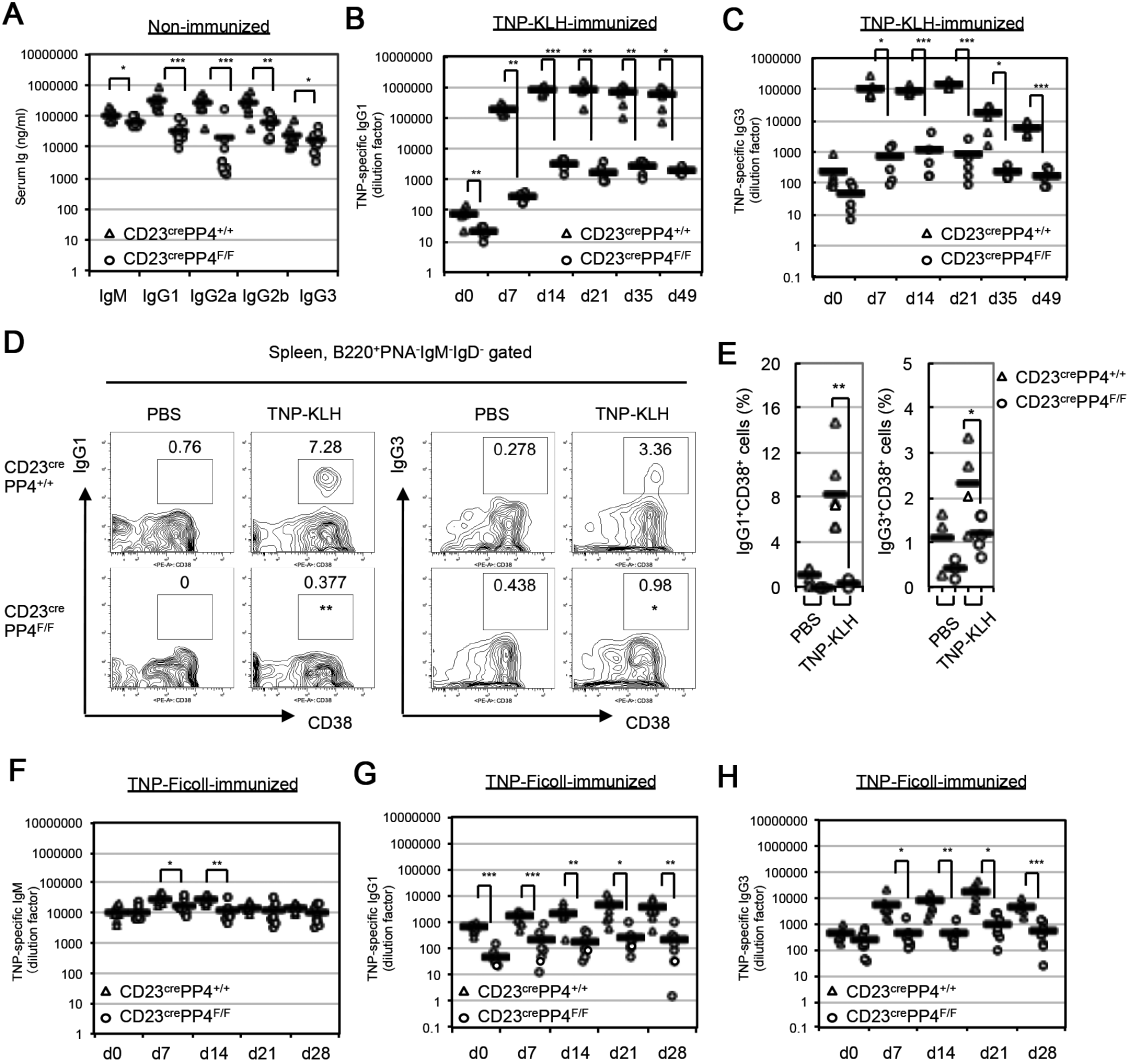
B cell-specific PP4 deficiency reduces Ig production *in* *vivo*. (**A**) Quantitation of serum levels of the indicated classes of Igs in non-immunized WT and CD23^cre^PP4^F/F^ mice (n = 8–10/group). Data are values for individual mice, and horizontal bars are geometric means. Results shown are from one experiment. (**B, C**) Quantitation of serum levels of TNP-specific IgG_1_ (**B**) and IgG_3_ (**C**) in WT and CD23^cre^PP4^F/F^ mice (n = 5–6/group) before immunization (d0), or at days 7, 14, 21, 35 and 49 post-immunization with TNP-KLH. Data are presented as in (A) and are from one experiment. (**D**) FACS profiles of CD38 vs IgG_1_ and CD38 vs IgG_3_ expression by B220^+^PNA^−^IgM^−^IgD^−^ gated splenocytes isolated from WT or CD23^cre^PP4^F/F^ mice (n = 3–6/group) at day 7 post-injection of PBS (control; left panels) or TNP-KLH (right panels). (**E**) Quantitation of the percentages of IgG_1_^+^ (left) and IgG_3_^+^ (right) switched B cells among total B cells from the mice in (D). Data are values for individual mice, and horizontal bars are geometric means. For (D–E), results are representative of two independent experiments. (**F–H**) Quantitation of serum levels of TNP-specific IgM (**F**), IgG_1_ (**G**) and IgG_3_ (**H**) in WT and CD23^cre^PP4^F/F^ mice (n = 8/group) before immunization (d0), or at days 7, 14, 21 and 28 post-immunization with TNP-Ficoll. Data are presented as in (A) and are from one experiment.

To investigate the role of PP4 in Ig class switching *in* *vivo*, we determined basal serum levels of IgM, IgG_1_, IgG_2a_, IgG_2b_ and IgG_3_ in WT and CD23^cre^PP4^F/F^ mice by ELISA. Significant decreases in all Ig isotypes were observed in the mutants (Figure 3A). When WT and CD23^cre^PP4^F/F^ mice were immunized with the Td antigen TNP-KLH, serum levels of TNP-specific IgG_1_ and IgG_3_ were drastically reduced in the mutants compared to WT controls (Figure 3B, 3C). In spleens of immunized WT mice, 7% of all B cells were IgG_1_^+^CD38^+^ and 3% were IgG_3_^+^CD38^+^, indicating successful class switching (Figure 3D, 3E). In contrast, negligible numbers of such Ig-switched B cells could be detected in CD23^cre^PP4^F/F^. Only a modest decrease in serum levels of TNP-specific IgM was observed in immunized mutant mice, and this only at day 7 post-immunization (Figure S3B).

Immunization of WT and CD23^cre^PP4^F/F^ mice with the Ti type II immunogen TNP-Ficoll also revealed a requirement for PP4 in CSR. Serum levels of TNP-specific IgM were reduced in immunized CD23^cre^PP4^F/F^ mice compared to WT controls at days 7 and 14 post-immunization, but reached to WT levels by days 21 and 28 post-immunization (Figure 3F). As expected, serum titers of TNP-specific IgG_1_ and IgG_3_ were significantly reduced in the immunized mutants compared to WT controls (Figure 3G, 3H). These data indicate that PP4 deficiency severely impairs *in* *vivo* Ig CSR in mice immunized with either a Td or Ti antigen.

### PP4 deficiency impairs germinal center (GC) induction

**Figure 4:**
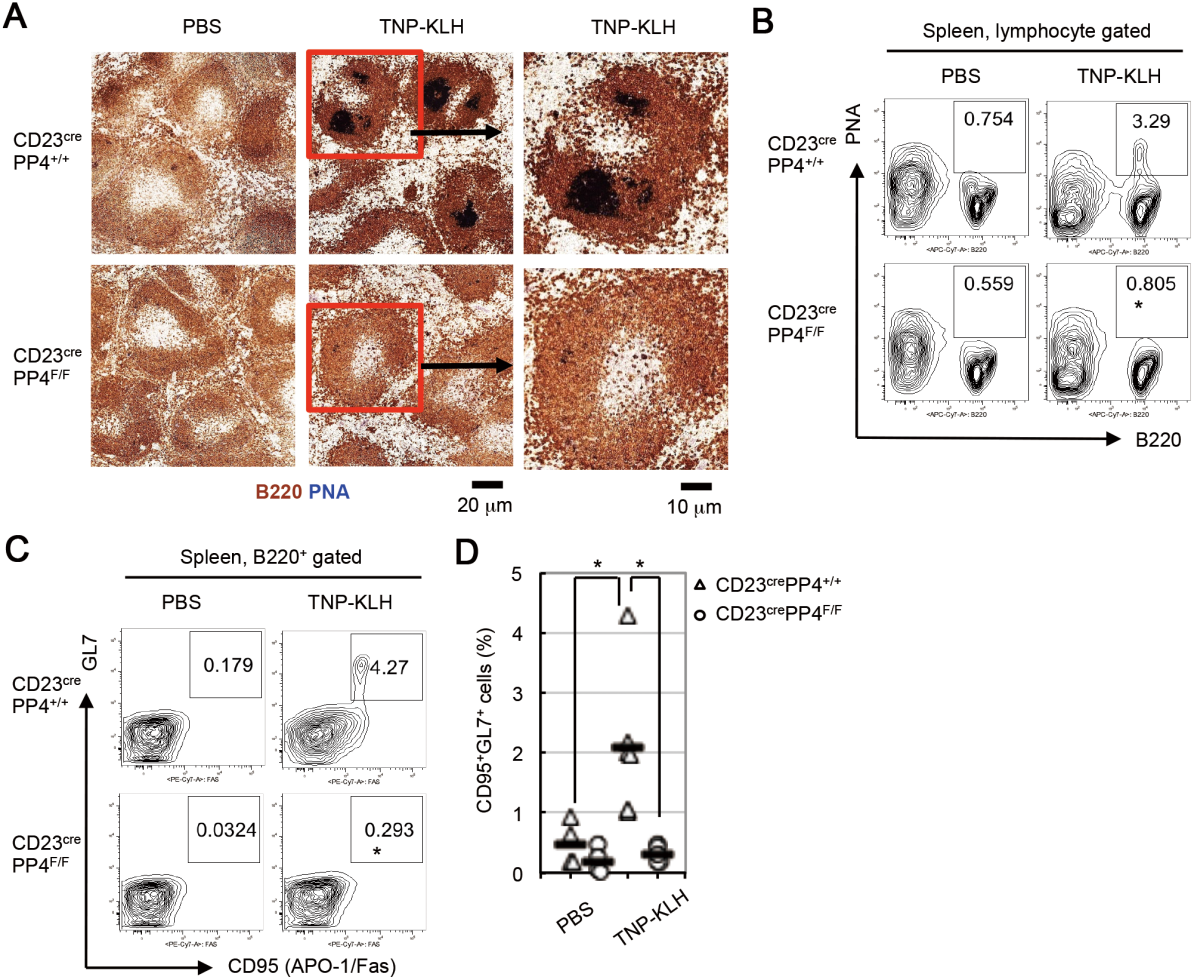
Impaired GC formation in CD23^cre^PP4^F/F^ mice immunized with TNP-KLH. (**A**) Immunohistochemical (IHC) images of PNA (dark blue) vs B220 (brown) expression in spleen sections of WT and CD23^cre^PP4^F/F^ mice (n = 3–6/group) at day 7 post-injection of PBS (control) or TNP-KLH. Data in the right panels are higher magnification images of the red rectangular areas in the center panels. (**B**) FACS profiles of B220 vs PNA expression by splenocytes from the mice in (A) at day 7 post-injection of PBS or TNP-KLH. (**C**) FACS profiles of CD95 vs GL7 expression by B220^+^-gated splenocytes from the mice in (A) at day 7 post-injection of PBS or TNP-KLH. (**D**) Quantitation of the percentage of GL7^+^CD95^+^ GC B cells among total B cells from the data in (C). Data are values for individual mice, and horizontal bars are geometric means. For (A–C), results are representative of two independent experiments.

To study whether PP4 deficiency affected GC B cell differentiation induced by immunization, WT and CD23^cre^PP4^F/F^ mice were i.p. injected with PBS (control) or the Td antigen TNP-KLH. At day 14 post-immunization, spleen sections were subjected to IHC analysis to detect GC formation. While B220^+^PNA^+^ GC B cells were clearly apparent in TNP-KLH-immunized WT mice, no GC B cells could be detected in immunized mutant mice (Figure 4A). FACS analysis revealed that 3.2% of total splenic B cells from immunized WT mice were PNA^+^B220^+^ GC B cells, while these cells represented less than 1% of total splenic B cells in immunized mutants (Figure 4B). Staining with anti-GL7 versus anti-CD95 Abs showed that 4.2% of total splenic B cells from immunized WT mice were GL7^+^CD95^+^ GC B cells, whereas GL7^+^CD95^+^ GC B cells were undetectable in spleens of immunized mutant mice (Figure 4C, 4D).

**Figure 5:**
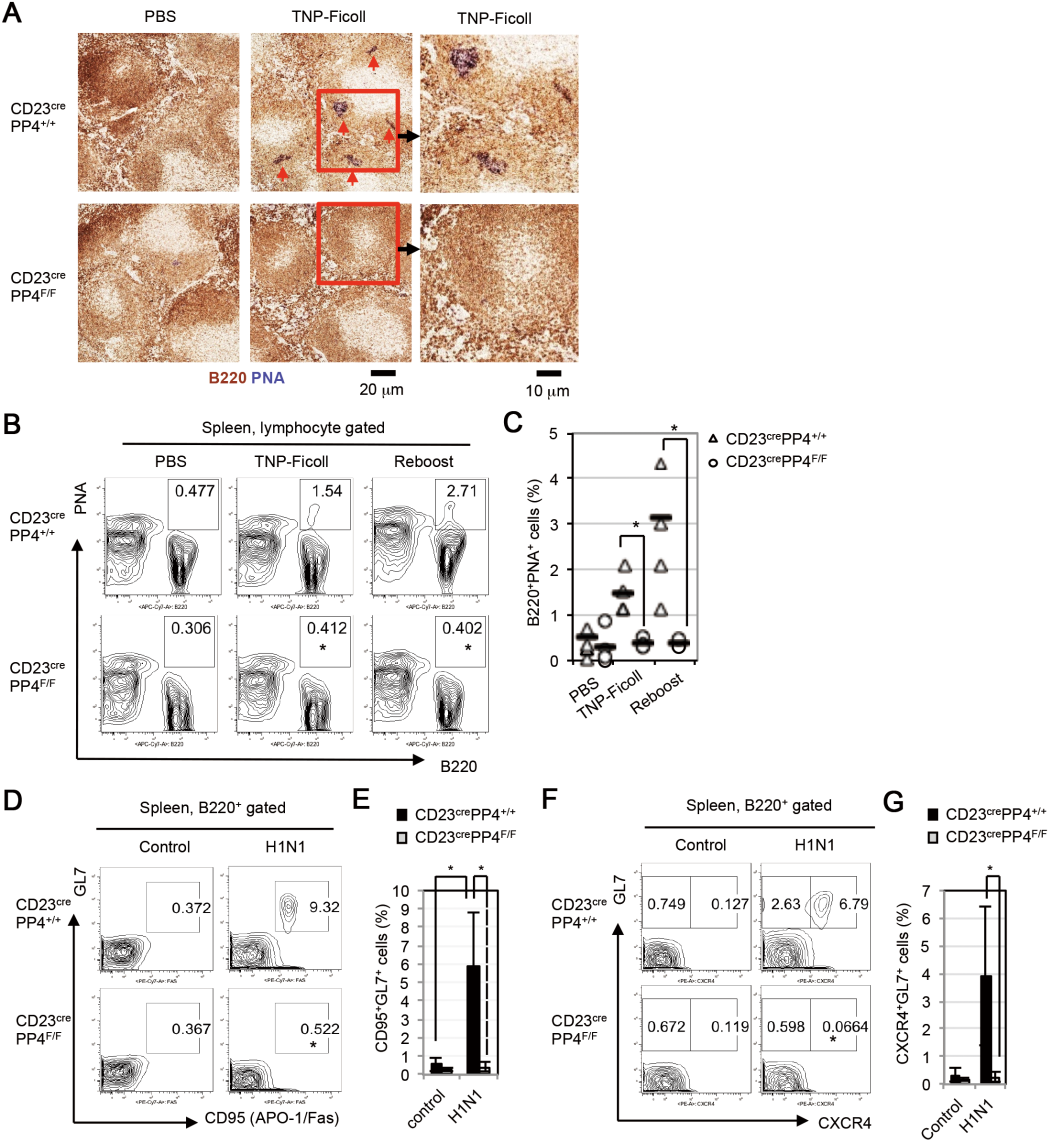
Impaired GC formation in CD23^cre^PP4^F/F^ mice immunized with TNP-Ficoll or infected with H1N1 virus. (**A**) IHC images of PNA (dark blue) vs B220 (brown) expression in spleen sections of WT and CD23^cre^PP4^F/F^ mice (n = 3–4/group) at day 14 post-injection of PBS or TNP-Ficoll. Data in the right panels are higher magnification images of the red rectangular areas in the center panels. Red arrows indicate GCs. (**B**) FACS profiles of B220 vs PNA expression in total splenocytes of the mice in (A). TNP-Ficoll-immunized WT and CD23^cre^PP4^F/F^ mice (n = 3–4/group) from those in (A) that were given a second injection of TNP-Ficoll at day 100 (Re-boost). (**C**) Quantitation of the percentage of PNA^+^B220^+^ GC B cells among total splenocytes of the mice in (B). Data are values for individual mice, and horizontal bars are geometric means. (**D**) FACS profiles of CD95 vs GL7 expression by B220^+^ splenic B cells isolated from WT and CD23^cre^PP4^F/F^ mice (n = 6/group) at day 15 post-injection of PBS or H1N1 virus. (**E**) Quantitation of the percentage of GL7^+^CD95^+^ GC B cells among total B cells from the data in (D). (**F**) FACS profiles of CXCR4 vs GL7 expression by B220^+^ splenic B cells from the mice in (D). (**G**) Quantitation of the percentage of GL7^+^CXCR4^+^ centroblasts among total B cells from the data in (F). For (A–G), results are representative of two independent experiments.

Because it was previously reported that GCs can be induced in response to TNP-Ficoll immunization [40], [41], we i.p. injected WT and CD23^cre^PP4^F/F^ mice with PBS or TNP-Ficoll. At day 14 post-immunization, a small number of PNA^+^B220^+^ GC B cells could be detected in WT mice by IHC, but no such GC B cells were present in immunized mutant mice (Figure 5A). FACS analysis confirmed that B220^+^PNA^+^ GC B cells constituted ∼1.5% of total splenocytes in immunized WT mice but were absent from immunized mutants (Figure 5B, 5C). Immunization with a second dose of TNP-Ficoll boosted GC B cells to 2.7% of total splenic B cells in WT mice but failed to induce the appearance of any GC B cells in mutant spleens (Figure 5B, 5C).

To determine if this defect in GC formation in CD23^cre^PP4^F/F^ mice had pathophysiological relevance, we infected WT and CD23^cre^PP4^F/F^ mice intranasally with a sub-lethal dose of the PR/8 strain of H1N1 influenza virus. At days 0 and 15 post-infection, spleen samples were harvested and subjected to FACS analysis. In infected WT mice, a significant induction of GL7^+^CD95^+^ GC B cells had occurred by day 15 post-infection (Figure 5D, 5E). In contrast, no GC B cells were generated in the spleens of infected mutant mice (Figure 5D, 5E). Furthermore, no GL7^+^CXCR4^+^ centroblasts could be detected in infected CD23^cre^PP4^F/F^ mice, whereas centroblasts constituted ∼2–6% of total splenic B cells in infected WT mice (Figure 5F, 5G). A failure to induce the generation of switched IgG_1_^+^ and IgG_3_^+^ B cells post-H1N1 infection was also observed in CD23^cre^PP4^F/F^ mice (Figure S3C–3E). In the draining mediastinal lymph nodes of WT mice, GC B cells and centroblasts were successfully induced at day 9 post-H1N1 infection, but not in those of infected mutant mice (Figure S4A–4D). As expected, serum titers of H1N1 Hemagglutinin-specific IgG_1_ and IgG_2a_ were significantly reduced in the infected mutants compared to WT controls (Figure S4E). Thus, PP4 deficiency prevents the differentiation of GC B cells induced by immunization with TNP-KLH or TNP-Ficoll, or upon H1N1 infection.

### PP4 deficiency impairs Ig CSR *in* *vitro*

**Figure 6:**
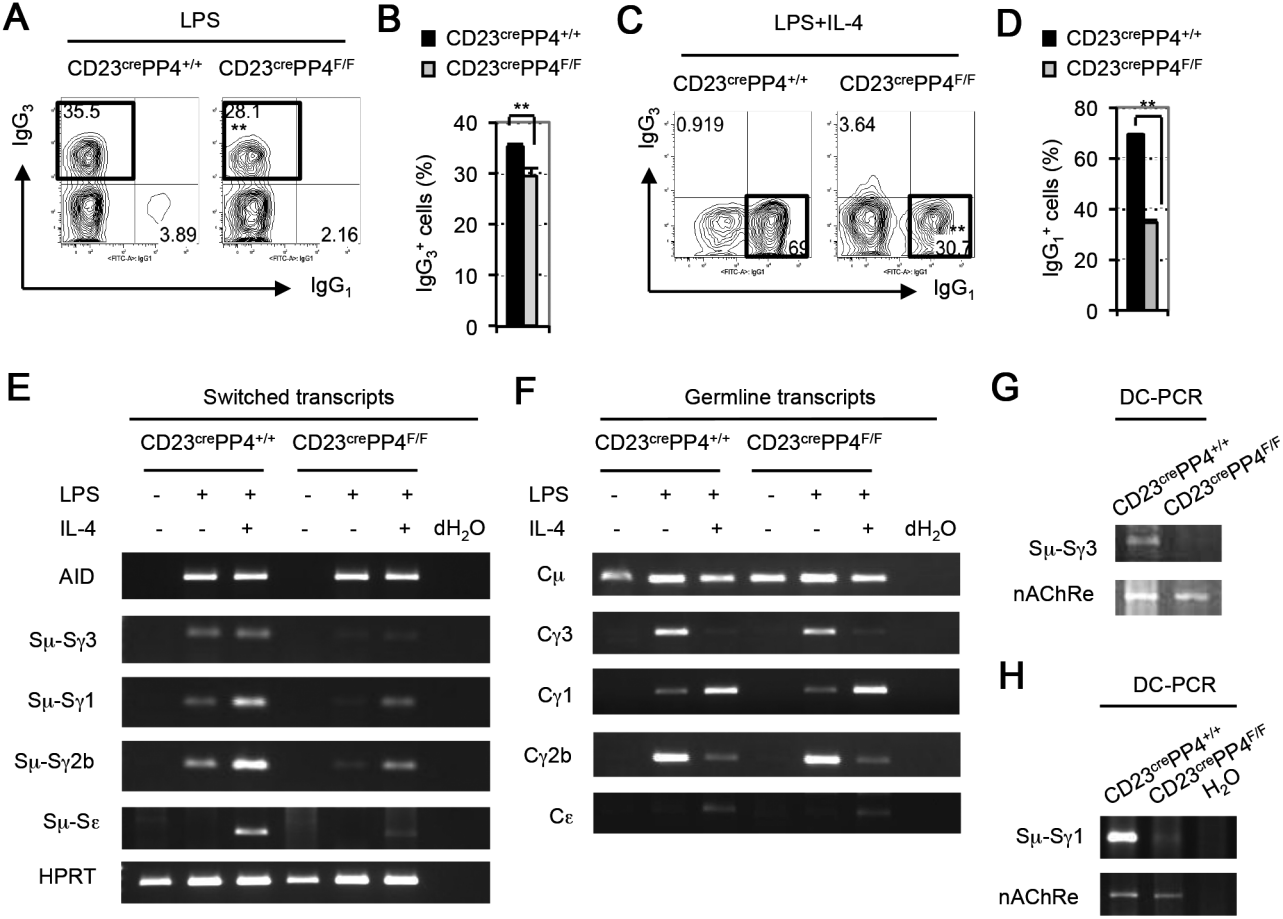
Impaired Ig class switching in PP4-deficient B cells. (**A**) FACS profiles of IgG_1_ vs IgG_3_ expression by B220^+^IgM^−^IgD^−^ gated B cells that were isolated from WT and CD23^cre^PP4^F/F^ mice (n = 3/group) and stimulated *in* *vitro* with LPS for 4 days. (**B**) Quantitation of the percentage of IgG_3_^+^-switched B cells among total B cells from the data in (A). Data are the mean ± SD of triplicates. For (A–B), results are representative of two independent experiments. (**C**) FACS profiles of IgG_1_ vs IgG_3_ expression by B220^+^IgM^−^IgD^−^-gated B cells that were isolated from WT and CD23^cre^PP4^F/F^ mice (n = 3/group) and stimulated *in* *vitro* with LPS plus IL-4 for 4 days. Data were analyzed as for (A). (**D**) Quantitation of the percentage of IgG_1_^+^-switched B cells among total B cells from the data in (C). For (C–D), results are representative of two independent experiments. (**E**) RT-PCR analysis of mRNA from B cells that were isolated from WT and CD23^cre^PP4^F/F^ mice (n = 4/group), and either left unstimulated or stimulated *in* *vitro* with LPS or LPS plus IL-4 for 4 days. Samples were analyzed to detect AID and the indicated switched transcripts. HPRT, loading control. (**F**) RT-PCR analysis of the indicated germline transcripts in the B cells in (E). Loading control was the same as in (E). For (E–F), results are representative of two independent experiments. (**G**) DC-PCR analysis of genomic DNA prepared from resting B cells isolated from WT and CD23^cre^PP4^F/F^ mice (n = 4/group). Genomic DNA was digested with *Eco*RI, ligated and utilized as template for PCR to detect the recombined Sµ-Sγ3 sequence (see Methods). nAChRe, loading control. (**H**) DC-PCR analysis of genomic DNA prepared from WT and CD23^cre^PP4^F/F^ B cells that were stimulated *in* *vitro* with LPS plus IL-4 for 4 days. The recombined Sµ-Sγ1 sequence was detected as illustrated in Figure S1. For (G–H), results are representative of two independent experiments.

It has been well established that CSR converting IgM to IgG_3_ can be triggered in murine B cells *in* *vitro* by LPS treatment, whereas a switch from IgM to IgG_1_ occurs following incubation with LPS plus IL-4 [42]. These *in* *vitro* conditions induce CSR in a manner that does not require GC formation, a fact that allowed us to study the effect of PP4 deficiency exclusively on CSR. Accordingly, we cultured total splenic B cells from WT or CD23^cre^PP4^F/F^ mice in the presence of LPS or LPS plus IL-4 for 4 days and monitored CSR. Upon stimulation with LPS alone, 35% of WT B cells switched to IgG_3_ production, compared to 29% of LPS-treated mutant B cells (Figure 6A). This decrease was modest but statistically significant (Figure 6B). After LPS+IL-4 stimulation, 68% of WT B cells produced IgG_1_, whereas only 30% of mutant B cells treated with LPS+IL-4 made the switch to IgG_1_ production (Figure 6C, 6D). The reduced switched-IgG_3_^+^ cells and IgG_1_^+^ cells by PP4 deficiency in percentages were associated with reduced switched cells in number (Figure S3F). To understand the dose effect of LPS in CSR, we compared IgG_3_- and IgG_1_-switching cells between WT and mutant B cells by various doses of LPS. We found that the numbers of IgG_3_^+^ and IgG_1_^+^ switching cells were remarkably reduced by PP4 deficiency in response to 50 ng/ml LPS, as well as 50 µg/ml LPS (Figure S3G, 3H). This dramatic decrease in switching to IgG production suggests that PP4 deficiency reduces CSR efficiency in a GC-independent manner.

During CSR, DNA breaks are introduced into both the donor IgM switch (S) region Sµ and a downstream S regions, followed by the joining of the broken donor Sµ site to the chosen acceptor S region. Successful joining is a prerequisite for the production of class-switched Ig transcripts and proteins. The reduced CSR efficiency we observed in our CD23^cre^PP4^F/F^ mice prompted us to investigate whether this deficit was associated with a decrease in switched Ig transcripts. To this end, we used a previously described protocol [34] to isolate total mRNA from WT and CD23^cre^PP4^F/F^ B cells cultured *in* *vitro* in the presence of LPS or LPS+IL-4, and subjected this mRNA to RT-PCR to determine levels of switched Ig transcripts. Strong induction of transcripts containing switched Sµ-Sγ3, Sµ-Sγ1, Sµ-Sγ2b and Sµ-Sε regions was observed in WT B cells treated with LPS or LPS+IL-4, but only low levels of these switched transcripts were detected in similarly-treated mutant B cells (Figure 6E). However, the induction of activation-induced cytidine deaminase (AID), the molecule that mediates CSR, was unchanged. In addition, levels of germline transcripts containing Cγ3, Cγ1, Cγ2b and Cε sequences were comparable in WT and mutant B cells (Figure 6F). These data support our hypothesis that PP4 deficiency impairs the production of switched Ig transcripts.

To determine whether the observed reduction in switched Ig transcripts in the mutant had occurred at the DNA level, we extracted genomic DNA from WT and CD23^cre^PP4^F/F^ B cells and used DC-PCR (see Methods and Figure S1A–1D) to amplify the switched sequences Sµ-Sγ3 and Sµ-Sγ1. Indeed, recombined Sµ-Sγ3 and Sµ-Sγ1 sequences were reduced in CD23^cre^PP4^F/F^ B cells compared to WT B cells (Figure 6G, 6H). To rule out the possibility that this decrease was due to the loss of PP4’s known negative regulation of LPS-induced NFκB-activation [17], we examined LPS-induced IκBα degradation in WT and CD23^cre^PP4^F/F^ B cells but found no abnormalities in the mutant cells (Figure S3I). These results therefore indicate that the reductions in CSR and switched Ig transcripts associated with PP4 deficiency are derived from impaired recombination of the Ig S regions.

### Increased DNA damage in PP4-deficient B cells

PP4 has been previously shown to regulate the DNA damage response [6]–[9]. To examine whether PP4 deficiency was associated with DNA damage in primary B cells, we subjected freshly isolated WT and CD23^cre^PP4^F/F^ splenic B cells to comet assays. As shown in Table 1, for all the parameters used to evaluate DNA damage, including tail intensity (tail % DNA), tail length, and Olive Tail Moment, scores were higher in mutant B cells than in WT B cells. These results demonstrate that PP4-deficient B cells exhibit increased DNA fragmentation even in the resting state, and suggest that loss of PP4 results in severe DNA damage.

**Table 1:** Comparison of comet assay scores between B cells from WT and CD23^cre^PP4^F/F^ mice.

B cells	Tail % DNA	Tail Length	Olive Tail Moment
CD23^cre^PP4^+/+^ (n = 452)	43.695633±18.2599	97.3738±21.8347	19.4059±10.9004
CD23^cre^PP4^F/F^ (n = 500)	56.7839±19.3081	102.1620±19.7717	27.1138±13.4265
*p*	1.6875E-25	0.0004	1.6529E-21

Tail % DNA, tail DNA expressed as a percentage of the comet’s total DNA [Tail % DNA = 100-head % DNA]; Tail Length, the horizontal distance from the end of the head to the end of the tail; Olive Tail Moment, the product of the tail length and the fraction of total DNA in the tail [Olive Tail Moment = (Tail mean−Head mean)×Tail % DNA/100]. B cells were a mixed population pooled from four mice per group. Results are representative of two independent experiments.

**Figure 7:**
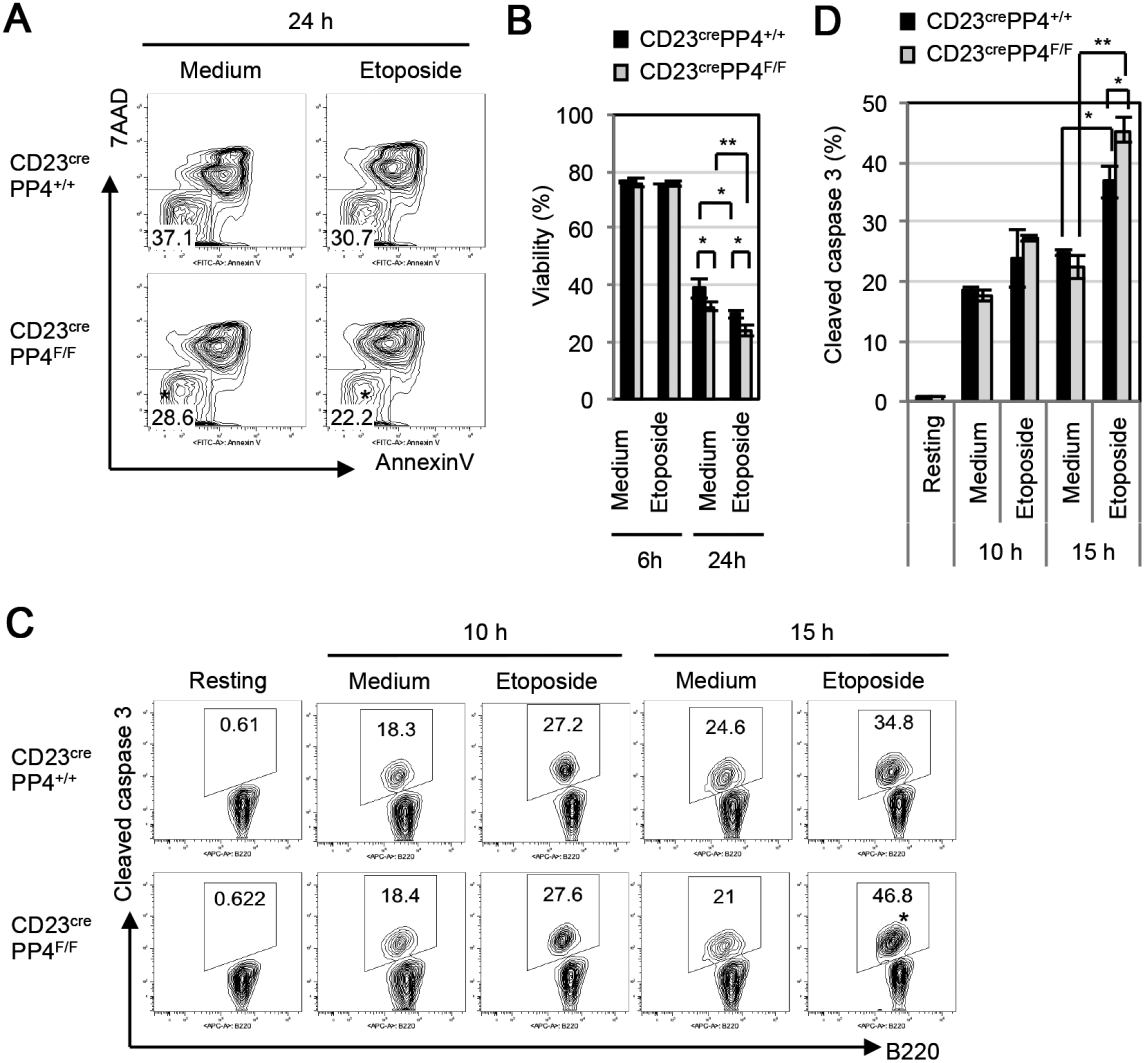
PP4-deficient B cells exhibit increased etoposide-induced caspase 3 cleavage. (**A**) FACS profiles of AnnexinV vs 7AAD staining of WT and CD23^cre^PP4^F/F^ B cells that were cultured for 6 h (not shown) or 24 h in maintenance medium alone or in maintenance medium containing 1 µM etoposide. (**B**) Quantitation of the percentage of viable (AnnexinV^−^7AAD^−^) cells among total B cells from the data in (A). Data are the mean ± SD of three mice per group. For (A–B), results are representative of two independent experiments. (**C**) FACS profiles of B220 vs cleaved caspase 3 staining of WT and CD23^cre^PP4^F/F^ B cells in the resting state (Resting), cultured in maintenance medium alone (Medium) or in maintenance medium containing 1 µM etoposide for 10 h or 15 h. (**D**) Quantitation of the percentage of B cells exhibiting cleaved caspase 3 among total B cells from data in (C). Data are the mean ± SD of three mice per group. For (C–D), results are representative of two independent experiments.

Despite the presence of increased DNA damage in CD23^cre^PP4^F/F^ B cells, our analyses of freshly isolated B cells showed that there was no increase in apoptosis in resting mature B cells in CD23^cre^PP4^F/F^ mice (Figure 2A, 2B). However, after growth *in* *vitro* for 24 h, cultures of mutant B cells exhibited a 5–6% reduction in viable (AnnevinV^−^7AAD^−^) cells (Figure 7A, 7B). When mutant B cells were treated *in* *vitro* with etoposide for 24 h, they again exhibited a 5–6% greater drop in viability compared to etoposide-treated WT B cells (Figure 7A, 7B). These data suggest that PP4-deficient B cells are sensitized to apoptosis induced by DNA-damaging drugs. Consistent with this hypothesis, CD23^cre^PP4^F/F^ B cells that were stimulated for 15 h with etoposide showed an increased level of cleaved caspase 3 compared to WT controls (Figure 7C, 7D). Thus, a deficiency of PP4 sensitizes cells to apoptosis *in* *vitro,* whether this apoptosis occurs in the absence of exogenous stimuli, or is triggered by DNA-damaging drugs.

### Impaired CD40 and BCR signaling, and impaired BCR/CD40-mediated S phase entry in PP4-deficient B cells

**Figure 8:**
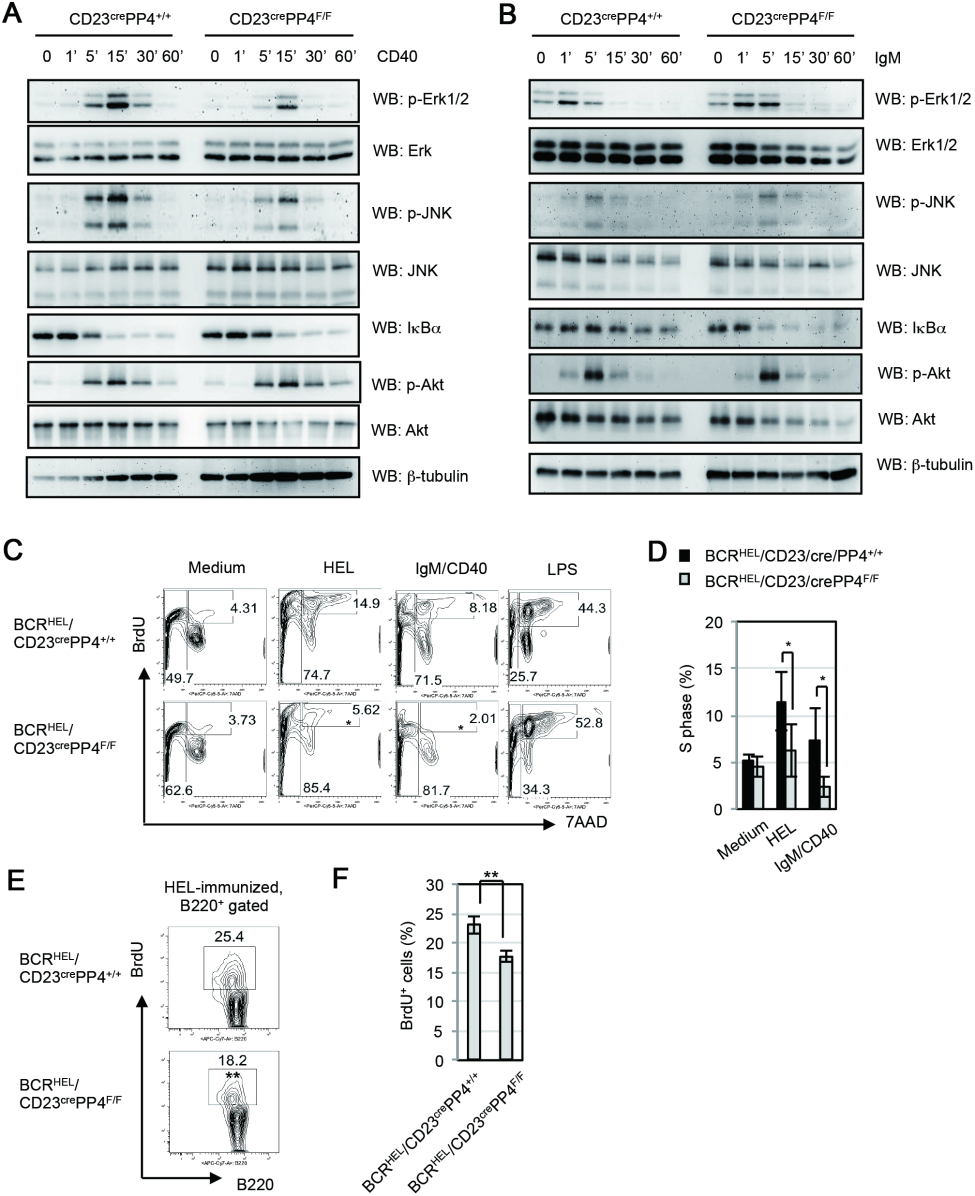
PP4-deficient B cells exhibit reduced CD40-mediated ERK and JNK activation, abnormal BCR-mediated IκBα degradation and reduced BCR/CD40-mediated S phase entry. (**A**) Western blot (WB) analysis of splenic B cells that were isolated from WT and CD23^cre^PP4^F/F^ mice (n = 6/group) and left unstimulated (0), or stimulated *in* *vitro* with anti-CD40 Ab for the indicated times. Lysates were subjected to WB to detect p-Erk1/2 (T202/Y204), total Erk, p-JNK (T183/Y185), total JNK, IκBα, p-Akt/PKB (S473), and total Akt. Results are representative of three independent experiments. (**B**) WB analysis of splenic B cells that were isolated from WT and CD23^cre^PP4^F/F^ mice (n = 6/group) and left unstimulated (0), or stimulated with anti-IgM Ab for the indicated times. Lysates were analyzed as in (A). Results are representative of two independent experiments. (**C**) FACS profile of *in* *vitro* BrdU incorporation assay. Splenic B cells were freshly isolated from BCR^HEL^/CD23^cre^PP4^+/+^ (n = 3) and BCR^HEL^/CD23^cre^PP4^F/F^ (n = 3) mice and cultured in BrdU-containing maintenance medium alone (Medium), or in maintenance medium containing 1 µg/ml HEL, 1 µg/ml anti-IgM plus anti-CD40 Abs (IgM/CD40) and 0.5 µg/ml LPS (LPS low). After 72 h, cells were analyzed by FACS. Numbers in upper quadrants represent the percentage of B cells in S phase relative to the total B cells after 72 h stimulation, while numbers in left quadrants represent the percentage of apoptotic B cells. (**D**) Quantitation of the mean percentage ± SD of B cells in S phase from the data in (C). β-tubulin, loading control. Results are representative of two independent experiments. (**E**) FACS profile of *in* *vivo* BrdU incorporation assay. BCR^HEL^/CD23^cre^PP4^+/+^ (n = 4) and BCR^HEL^/CD23^cre^PP4^F/F^ (n = 4) mice were immunized with HEL in alum at day 0 and injected with BrdU from day 3 in 4 consecutive days as illustrated in Figure S5A. At day 7 after immunization, splenic B cells were harvested and analyzed by FACS. Numbers in quadrants represent the percentage of B cells in S phase (BrdU^+^) relative to total B cells. Results are representative of two independent experiments. (**F**) Quantitation of the mean percentage ± SD of B cells in S phase from the data in (E).

CD40 engagement is crucial for the GC reaction, and CD40-transduced MAPK activation is required for GC formation and B cell antibody production [43]. To examine PP4’s function in CD40-mediated signaling events, WT and CD23^cre^PP4^F/F^ splenic B cells were left unstimulated, or stimulated *in* *vitro* with anti-CD40 Ab for up to 60 min. In WT B cells, maximum phosphorylation of Erk1/2 and JNK was achieved after 15 min of anti-CD40 stimulation. In mutant B cells, although the kinetics and patterns of Erk1/2 and JNK phosphorylation were similar to those in WT B cells, the strength of these phosphorylation events was weaker than in controls (Figure 8A). Akt/PKB phosphorylation and IκBα degradation induced by CD40 engagement were comparable in kinetics, patterns and strength in WT and mutant B cells. Interestingly, the reductions in Erk1/2 and JNK phosphorylation observed in anti-CD40-stimulated mutant B cells did not occur when these cells were stimulated by anti-IgM Ab (Figure 8B). Nevertheless, the kinetics of IκBα degradation mediated by BCR-stimulation in mutant B cells occurred much faster than WT B cells. The result was similar to the earlier finding, which identified PP4 complex as a negative regulator of NF-κB activation mediated by T cell receptor signaling [20]. Our data therefore reveal a specific role for PP4 in ensuring optimum CD40-mediated MAPK activation and BCR-mediated NF-κB activation in primary B cells.

Despite the essential role of CD40 signaling in GC formation, we could not induce cells into S phase in WT B cells by CD40-stimulation or BCR/CD40-stimulation for 48 h (Fig. 2E). We assume that B cells of huge heterogeneity in BCR might be one of reasons responsible for a poor proliferating effect. To address whether the defect in GC formation in CD23^cre^PP4^F/F^ mice is associated with impaired BCR/CD40-mediated proliferation, we crossed CD23^cre^PP4^F/F^ mice with BCR^HEL^ transgenic mice to generate BCR^HEL^/CD23^cre^PP4^F/F^ mice [33]. B cells from BCR^HEL^ transgenic mice carry BCRs recognizing hen egg lysozyme (HEL). BCR^HEL^/CD23^cre^PP4^+/+^ mice were bred and B cells from these mice were utilized as control. Splenic B cells from BCR^HEL^/CD23^cre^PP4^+/+^ mice and BCR^HEL^/CD23^cre^PP4^F/F^ mice were purified, left unstimulated, or stimulated with HEL, anti-IgM plus anti-CD40 (IgM/CD40), or LPS for 72 h in full medium supplemented with BrdU, followed by the subjection to BrdU incorporation analysis. We found that 14% and 8% of transgenic WT B cells from BCR^HEL^/CD23^cre^PP4^+/+^ mice were in S phase upon HEL- and IgM/CD40-stimulation respectively. However, transgenic mutant B cells from BCR^HEL^/CD23^cre^PP4^F/F^ mice showed significant reductions of cells in S phase upon HEL- and IgM/CD40-stimulation (Fig. 8C, 8D). Our data therefore reveal that PP4 is essential for S phase entry mediated by IgM/CD40, which mimics GC activation *in* *vivo*. To address whether the reduced S phase-entry occurred in mutant mice upon immunization *in* *vivo*, we performed BrdU incorporation assay in BCR^HEL^/CD23^cre^PP4^+/+^ and BCR^HEL^/CD23^cre^PP4^F/F^ mice upon immunization with HEL in alum. At day 7 after immunization, GC B cells were induced in BCR^HEL^/CD23^cre^PP4^+/+^ mice but not in BCR^HEL^/CD23^cre^PP4^F/F^ mice (Figure S5A–5C). Under this condition, transgenic mutant B cells from BCR^HEL^/CD23^cre^PP4^F/F^ mice showed a slight but significant reduction of cells in S phase compared to transgenic WT B cells (Fig. 8E, 8F). In addition, the percentage of AnnexinV^−^7AAD^−^ viable B cells in transgenic mutant B cells was slightly but significantly less than those in transgenic WT B cells (Figure S5D, 5E). The findings support the hypothesis that the impaired GC formation in mutant mice *in* *vivo* was associated with a proliferation defect, which disabled antigen-specific B cells for the clonal expansion required for GC formation. Taken together, our findings establish crucial roles for PP4 in the maintenance of genomic stability, GC formation, and Ig class switching.

## Discussion

In this report, we used conditional mutant CD23crePP4^F/F^ mice to demonstrate that PP4 is specifically required for GC formation and CSR in mature B cells. This work builds on our previous study utilizing mb-1/cre-mediated PP4 ablation in B cell progenitors, which revealed that PP4 is critical for VDJ recombination and B cell development. Interestingly, PP4 deficiency in mature B cells resulted in a 20–50% reduction in the production of switched IgG1 and IgG3 transcripts in vitro, suggesting that CSR is less efficient in the absence of PP4. A previous study using a macrophage cell line showed that PP4 negatively regulates LPS-induced NFκB-activation [17]. Although LPS-stimulated PP4-deficient B cells showed increased S phase entry, our mutant B cells displayed no defect in either LPS-mediated IκBα degradation or the expression of costimulatory molecules relying on the NFκB-pathway. Moreover, LPS-stimulated PP4-deficient B cells produced WT levels of Cγ1, Cγ3 and Cγ2b germline transcripts in vitro. Thus, the reduced CSR in our PP4-deficient B cells was most likely due to inefficient joining of S regions rather than to impaired TLR4 signaling.

In this study, we also identified an important B cell-intrinsic function for PP4 in GC formation. When CD23^cre^PP4^F/F^ mice were immunized with TNP-KLH, the formation of GCs was not observed in the lymphoid follicles of these mutants. This finding suggests that the severe reduction in antigen-specific serum Igs observed in our mutant mice *in* *vivo* was not only due to inefficient CSR but also to a GC defect. During GC formation in WT mice, B cells expressing BCRs of high affinity for the inducing antigen are selectively expanded. Although the BCR is central to this B cell selection process, the precise BCR-mediated signaling cascade involved is not well understood. We observed impaired BCR-mediated S phase entry and a faster degradation of IκBα upon BCR-stimulation in the absence of PP4, defects that might disable the selection of B cells carrying high affinity BCRs. In addition, CD40-mediated phosphorylation of both ERK and JNK was reduced in PP4-deficient B cells, a finding consistent with a previous report showing that dominant negative mutation of PP4 can block TNF-α-induced JNK activation [15]. CD40-mediated JNK signaling is required to sustain GC formation [44], and so the loss of this capacity in PP4-deficient B cells could contribute to the phenotype of our CD23^cre^PP4^F/F^ mice. Intriguingly, previous work has shown that, although CD40-deficient mice show impaired CSR and GC formation, their production of antigen-specific IgM in response to immunization with Td or Ti antigens is normal [25]. In contrast, we found that TNP-specific IgM secretion was reduced in CD23^cre^PP4^F/F^ mice immunized with TNP-Ficoll or TNP-KLH. These data again revealed that PP4 deficiency has fundamental effects in CD40- and BCR-signaling pathways. Our finding of the impaired BCR/CD40-mediated S phase entry *in* *vitro* and the reduced S phase entry in response to HEL-immunization *in* *vivo* in transgenic B cells deficient of PP4 strongly suggests that the impaired GC formation in mutant mice derives from the proliferation defects.

In our previous study of mb-1/cre/PP4^F/F^ mice, we found that unrepaired DNA breaks in the Ig loci could be detected in B cells at the CD43^+^CD24^+^BP-1^−^ stage (Hardy fraction B) [21]. This failure in VDJ recombination resulted in severe DNA fragmentation that likely triggered apoptosis at the next B cell developmental stage (CD43^+^CD24^+^BP-1^+^, Hardy fraction C). In the present study, we again observed severe DNA fragmentation in CD23^cre^PP4^F/F^ B cells in the resting state. The mutant B cells were more sensitive to apoptosis induced by a DNA-damaging drug *in* *vitro*. When CD23^cre^PP4^F/F^ B cells were stimulated with etoposide for 15 h, an increase in caspase 3 cleavage was detected. These data imply that the genotoxic stress caused by PP4 deficiency may antagonizes signaling events required for GC formation and promotes the apoptosis of the defective cells.

The causes of the initial DNA fragmentation in resting PP4-deficient splenic B cells remain to be determined. Because the PP4 complex participates in multiple pathways involved in the DNA damage response [4], [5], [7], [9], it would be reasonable to conclude that PP4 deficiency results in an accumulation of DNA damage during homeostatic B cell expansion. In the absence of PP4, DNA damage in the mutant B cells would not be efficiently repaired. However, this deficit on its own would neither trigger immediate apoptosis nor impair the generation of FO and MZ B cells. It may be that the pre-existing DNA fragmentation present in PP4-deficient naïve B cells induces them to undergo apoptosis via the caspase pathway only upon exposure to exogenous stimuli or foreign antigen. Thus, the impaired CSR associated with PP4 deficiency may be derived not only from defects in the DNA repair system specifically involved in CSR, but also from a failure to counter the more general effects of genotoxic stress.

We have now demonstrated that PP4 is involved in both VDJ recombination and CSR in B cells, but how PP4 modulates the DNA repair events required for these processes is not yet clear. Our data indicates that the defect on CSR in vivo is likely to stem from the dramatic effect of PP4 deficiency on germinal centre formation. Nevertheless, it is not known whether the partial requirement for PP4 for CSR in vitro was accounted for by problems in DNA repair pathways involved in CSR, or the consequence of the genotoxic stress and/or the impaired proliferation. During B cell maturation and differentiation, cells undergoing VDJ recombination or CSR are in a state of genomic instability, in that DNA breaks are deliberately introduced which require intact repair systems to restore DNA functionality. Lesions occurring in the S regions during CSR are repaired by the NHEJ pathway and the alternative end-joining (A-EJ) pathway [22]. γH2AX and KAP-1 might be two factors involved in the NHEJ pathway, and PP4 has been shown to dephosphorylate them both [5]–[9]. In addition, 53BP1, which contributes to CSR, has been identified by phosphoproteomic studies as a putative PP4 substrate [8], [45]. PP4 deficiency could presumably result in the sustained phosphorylation of these or other unidentified substrates, interfering with normal CSR. Further investigation into the molecular mechanisms regulated by PP4 should yield valuable new insights into CSR regulation.

PP4 has been implicated in a wide variety of biological processes through its targeting of diverse substrates. Furthermore, emerging data suggest that PP4 might also participate in tumor formation [20], [46], [47]. PP4 is overexpressed in human breast cancer and lung cancer [46], and high levels of PP4 correlate with poor prognosis in patients with stage II pancreatic ductal adenocarcinoma [47]. We speculate that the genotoxicity we found to be associated with PP4 deficiency may increase the risk of mutagenesis in B cells, potentially contributing to lymphomagenesis. Further investigation of PP4 levels and functions in B cell-associated malignancies may help us to understand PP4’s clinical relevance. The present study has highlighted the basic physiological relevance of PP4 in genomic stability, GC formation and CSR, and has shown that inactivation of PP4 leads to immunodeficiency through impairment of both Td and Ti antibody responses.

## Supporting Information

Figure S1 **Schematic illustration of digestion-circularization (DC)-PCR to detect the switched Sµ-Sγ1 sequence.** (**A**) Schematic illustration of germline structure of Ig switch (S) regions and constant (C) regions. (**B**) Schematic illustration of the switched Sµ-Sγ1 structure. (**C, D**) For first-round DC-PCR (C), *Eco*RI-digested and re-ligated circular DNA was used as a template and primers “RBT43” (5′-GAGAGCAGGATCTCCTGGGTAGG-3′) and “sg1” (5′-AACAAAGTCACTGTAAATGCT TCGGGTA-3′) were employed (94°C 30″, 64°C 30″, 72°C 35″ for 15 cycles). For second-round nested PCR, primers “sg1” and “smu” (5′-TAGTTGGGGATTCTAAGCAGTCACAGA-3′) were used (94°C 30″, 61°C 30″, 72°C 20″ for 32 cycles). The generation of a PCR product of 242 bp indicated successful Sµ-Sγ1 recombination.
(PDF)

Figure S2 **Generation of CD23^cre^PP4^F/F^ mice and anti-PP4 antibody.** (**A**) Schematic illustration of the targeting strategy used to delete *pp4c*. The positions of the primers designed to assess deletion efficiency are indicated by black arrows. For genomic PCR, forward primers “f1” (5′-ACGTGATTTGCGAAAGCCTCTCA-3′) and “f2” (5′-CTTGGTAGAAGAGAGCAACGTGCAG-3′), and reverse primer “r” (5′-TGCCTGGTGGCAGGAGATGTGTG-3′), were employed as indicated. The PCR products of the WT *pp4c* allele (427 bp; upper panel); the *pp4c floxp* allele before cre-mediated deletion (480 bp; middle panel); and the *pp4c floxp* allele after cre-mediated deletion (550 bp; lower panel) are shown. (**B**) Western blot (WB) analysis using an in-house rabbit polyclonal anti-PP4 antibody (Ab) to detect PP4 in 293T cells overexpressing PP4-Flag fusion protein. (**C**) WB analysis using the in-house anti-PP4 Ab (upper panel) or anti-PP2Ac Ab (lower panel) to detect PP4 or PP2Ac in 293T cells overexpressing PP4-Flag or PP2Ac-HA fusion proteins, respectively.
(PDF)

Figure S3 **Various assays characterizing CD23^cre^PP4^F/F^ mice and their B cells.** (**A**) FACS profile of CD40 vs CD25 expression by WT and CD23^cre^PP4^F/F^ B cells that were left unstimulated (Medium), or stimulated for 48 h *in* *vitro* in maintenance medium containing 1 µg/ml anti-IgM or 10 µg/ml anti-IgM. (**B**) Serum levels of TNP-specific IgM in WT and CD23^cre^PP4^F/F^ mice (n = 8–10/group) on the indicated days post-immunization with TNP-KLH. Data are values for individual mice and horizontal bars are geometric means. Results shown are from one experiment. (**C**) FACS profile of CD38 vs IgG_1_ expression by gated B220^+^IgM^−^IgD^−^CD95^−^PNA^−^ splenic B cells from WT and CD23^cre^PP4^F/F^ mice at day 15 post-H1N1 infection. H1N1 #1 and #2 are identically infected mice in each group. Numbers in quadrants are the percentage of IgG_1_^+^-switched B cells among total B cells. (**D**) Quantitation of the percentage of IgG_1_^+^-switched B cells among total B cells from the data in (C). (**E**) Quantitation of the percentage of IgG_3_^+^-switched B cells among B220^+^IgM^−^IgD^−^CD95^−^PNA^−^-gated B cells from the data in (C). For (C–E), results are representative of two independent experiments. (**F**) Quantitation of the percentage of IgG_3_^+^- and IgG_1_^+^-switched B cells (gated from B220^+^IgM^−^IgD^−^ cells) among total B cells from the data in Figure 6A to 6D. (**G**) Quantitation of the percentage of IgG_3_^+^-switched B cells (gated from B220^+^IgM^−^IgD^−^ cells) among total B cells induced by various doses of LPS. (**H**) Quantitation of the percentage of IgG_1_^+^-switched B cells (gated from B220^+^IgM^−^IgD^−^ cells) among total B cells induced by various doses of LPS plus IL-4. (**I**) WB analysis of IκBα degradation in WT and CD23^cre^PP4^F/F^ B cells that were stimulated *in* *vitro* with 5 µg/ml LPS for the indicated times. gp96, loading control. Results are representative of two independent experiments.
(TIFF)

Figure S4 **Impaired immune responses in CD23^cre^PP4^F/F^ mice infected with H1N1 virus.** (**A**) FACS profiles of GL7 vs CD95 expression by B220^+^ lymphocytes isolated from the mediastinal lymph nodes in WT and CD23^cre^PP4^F/F^ mice (n = 4/group) at day 9 post-injection of PBS or H1N1 virus. (**B**) Quantitation of the percentage of GL7^+^CD95^+^ GC B cells among total B cells from the data in (A). (**C**) FACS profiles of GL7 vs CXCR4expression by B220^+^ lymphocytes isolated from the mediastinal lymph nodes in WT and CD23^cre^PP4^F/F^ mice (n = 4/group) at day 9 post-injection of PBS or H1N1 virus. (**D**) Quantitation of the percentage of GL7^+^CXCR4^+^ centroblasts among total B cells from the data in (C). For (A–D), results are representative of two independent experiments. (**E**) Quantitation of serum levels of H1N1-specific IgG_1_ and IgG_2a_ in WT and CD23^cre^PP4^F/F^ mice (n = 5–6/group) before infection (d0) or at day 9 post-infection with H1N1. Data are from one experiment.
(TIFF)

Figure S5 **Reduced cell proliferation and reduced viability in transgenic mutant B cells from BCR^HEL^CD23^cre^PP4^F/F^ mice with HEL immunization.** (**A**) Illustration of the experiment procedure with HEL-immunization. BCR^HEL^CD23^cre^PP4^+/+^ and BCR^HEL^CD23^cre^PP4^F/F^ mice (n = 4/group) were immunized with HEL in alum at day 0 and injected with BrdU from days 3 to 6. Mice were dissected at day 7 post-immunization and analyzed by FACS. (**B**) FACS profiles of PNA vs CD95 expression by B220^+^ splenocytes in BCR^HEL^CD23^cre^PP4^+/+^ and BCR^HEL^CD23^cre^PP4^F/F^ mice at day 7 after immunization. (**C**) Quantitation of the percentage of PNA^+^CD95^+^ GC B cells among total splenic B cells from the data in (B). (**D**) FACS profiles of AnnexinV vs 7AAD expression by B220^+^ splenocytes in BCR^HEL^CD23^cre^PP4^+/+^ and BCR^HEL^CD23^cre^PP4^F/F^ mice at day 7 after immunization. (**E**) Quantitation of the percentage of AnnexinV^−^7AAD^−^ viable B cells among total B cells from the data in (D).
(TIFF)
