# Supplementary material for: PP4 Is Essential for Germinal Center Formation and Class Switch Recombination in Mice
Source: PLoS One. 2014 Sep 12;9(9):e107505. doi: 10.1371/journal.pone.0107505 (PMC4162579; doi:10.1371/journal.pone.0107505)
Supplement: Figure S1 — Schematic illustration of digestion-circularization (DC)-PCR to detect the switched Sµ-Sγ1 sequence. (A) Schematic illustration of germline structure of Ig switch (S) regions and constant (C) regions. (B) Schematic illustration of the switched Sµ-Sγ1 structure. (C, D) For first-round DC-PCR (C), EcoRI-digested and re-ligated circular DNA was used as a template and primers “RBT43” (5′-GAGAGCAGGATCTCCTGGGTAGG-3′) and “sg1” (5′-AACAAAGTCACTGTAAATGCT TCGGGTA-3′) were employed (94°C 30″, 64°C 30″, 72°C 35″ for 15 cycles). For second-round nested PCR, primers “sg1” and “smu” (5′-TAGTTGGGGATTCTAAGCAGTCACAGA-3′) were used (94°C 30″, 61°C 30″, 72°C 20″ for 32 cycles). The generation of a PCR product of 242 bp indicated successful Sµ-Sγ1 recombination. (PDF) [file pone.0107505.s001.pdf]

Supporting figure 1

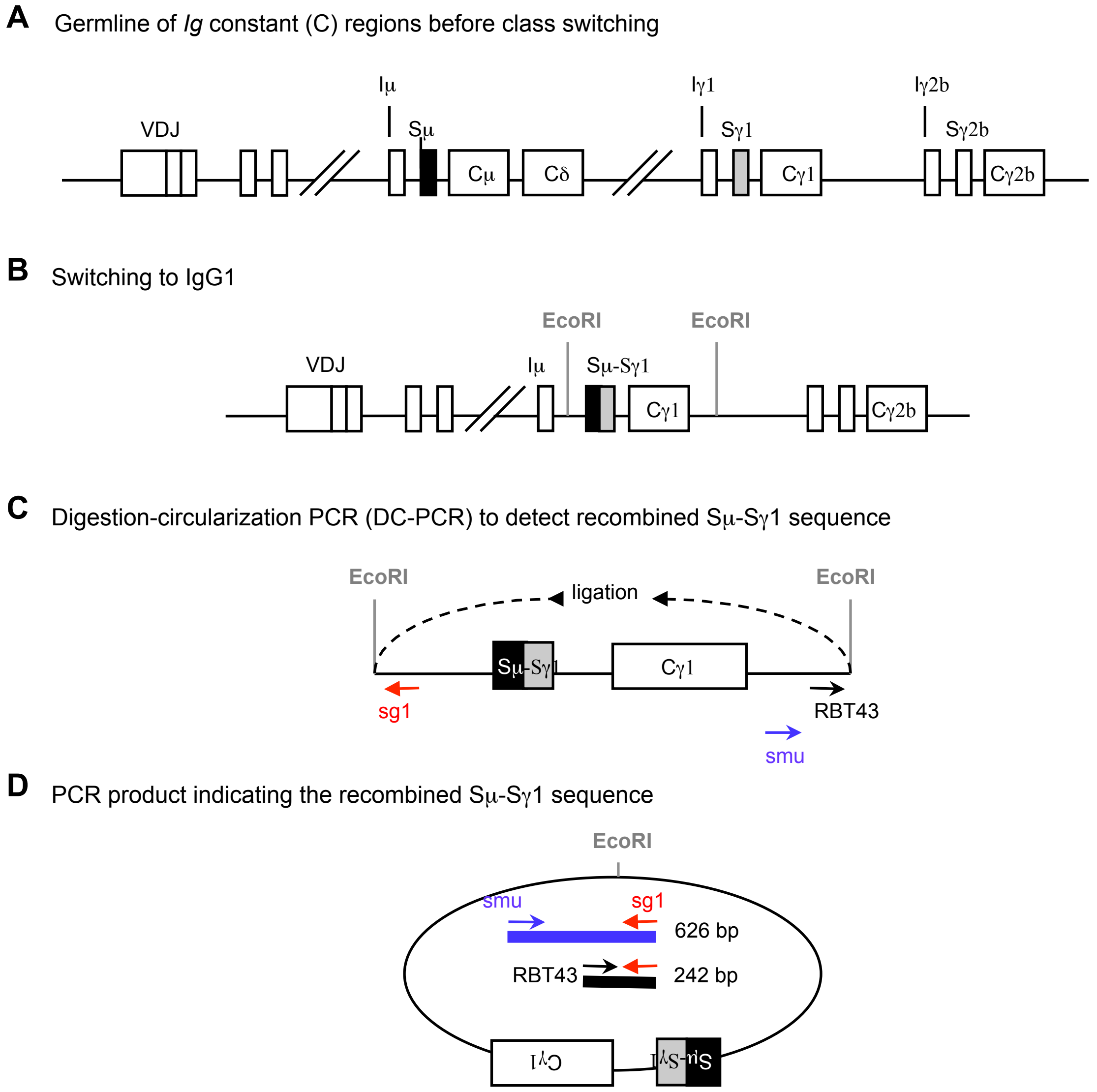

Schematic illustration of digestion-circularization (DC)-PCR to detect the switched Sμ-Sγ1 sequence. (A) Schematic illustration of germline structure of Ig switch (S) regions and constant (C) regions. (B) Schematic illustration of the switched Sμ-Sγ1 structure. (C, D) For first-round DC-PCR (C), EcoRI-digested and re-ligated circular DNA was used as a template and primers “RBT43” (5’-GAGAGCAGGATCTCCTGGGTAGG-3’) and “sg1” (5’-AACAAAGTCACTGT AAATGCTTCGGGTA-3’) were employed (94°C 30”, 64°C 30”, 72°C 35” for 15 cycles). For second-round nested PCR, primers “sg1” and “smu” (5’-TAGTTGGGGATTCTAAGCATCACA GA-3’) were used (94°C 30”, 61°C 30”, 72°C 20” for 32 cycles). The generation of a PCR product of 242 bp indicated successful Sμ-Sγ1 recombination.
