# Supplementary material for: PP4 Is Essential for Germinal Center Formation and Class Switch Recombination in Mice
Source: PLoS One. 2014 Sep 12;9(9):e107505. doi: 10.1371/journal.pone.0107505 (PMC4162579; doi:10.1371/journal.pone.0107505)
Supplement: Figure S2 — Generation of CD23crePP4F/F mice and anti-PP4 antibody. (A) Schematic illustration of the targeting strategy used to delete pp4c. The positions of the primers designed to assess deletion efficiency are indicated by black arrows. For genomic PCR, forward primers “f1” (5′-ACGTGATTTGCGAAAGCCTCTCA-3′) and “f2” (5′-CTTGGTAGAAGAGAGCAACGTGCAG-3′), and reverse primer “r” (5′-TGCCTGGTGGCAGGAGATGTGTG-3′), were employed as indicated. The PCR products of the WT pp4c allele (427 bp; upper panel); the pp4c floxp allele before cre-mediated deletion (480 bp; middle panel); and the pp4c floxp allele after cre-mediated deletion (550 bp; lower panel) are shown. (B) Western blot (WB) analysis using an in-house rabbit polyclonal anti-PP4 antibody (Ab) to detect PP4 in 293T cells overexpressing PP4-Flag fusion protein. (C) WB analysis using the in-house anti-PP4 Ab (upper panel) or anti-PP2Ac Ab (lower panel) to detect PP4 or PP2Ac in 293T cells overexpressing PP4-Flag or PP2Ac-HA fusion proteins, respectively. (PDF) [file pone.0107505.s002.pdf]

Supporting figure 2

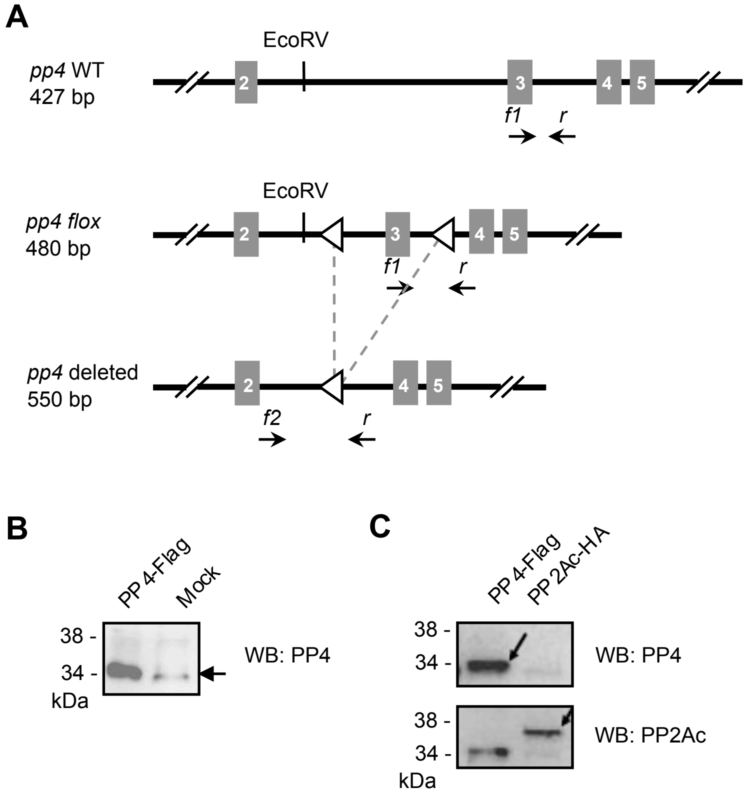

Generation of CD23^{cre}PP4$^{F/F}$ mice and anti-PP4 antibody. (**A**) Schematic illustration of the targeting strategy used to delete *pp4c*. The positions of the primers designed to assess deletion efficiency are indicated by black arrows. For genomic PCR, forward primers “f1” (5’-ACGTGA TTTGCGAAAGCCTCTCA-3’) and “f2” (5’-CTTGGTAGAAGAGA GCAACGTGCAG-3’), and reverse primer “r” (5’-TGCCTGGTGGCAGGAGATGTGTG-3’), were employed as indicated. The PCR products of the WT *pp4c* allele (427 bp; upper panel); the *pp4c floxp* allele before cre-mediated deletion (480 bp; middle panel); and the *pp4c floxp* allele after cre-mediated deletion (550 bp; lower panel) are shown. (**B**) Western blot (WB) analysis using an in-house rabbit polyclonal anti-PP4 antibody (Ab) to detect PP4 in 293T cells overexpressing PP4-Flag fusion protein. (**C**) WB analysis using the in-house anti-PP4 Ab (upper panel) or anti-PP2Ac Ab (lower panel) to detect PP4 or PP2Ac in 293T cells overexpressing PP4-Flag or PP2Ac-HA fusion proteins, respectively.
