# Supplementary figures and images for: PP4 Is Essential for Germinal Center Formation and Class Switch Recombination in Mice
Source: PLoS One. 2014 Sep 12;9(9):e107505. doi: 10.1371/journal.pone.0107505 (PMC4162579; doi:10.1371/journal.pone.0107505)

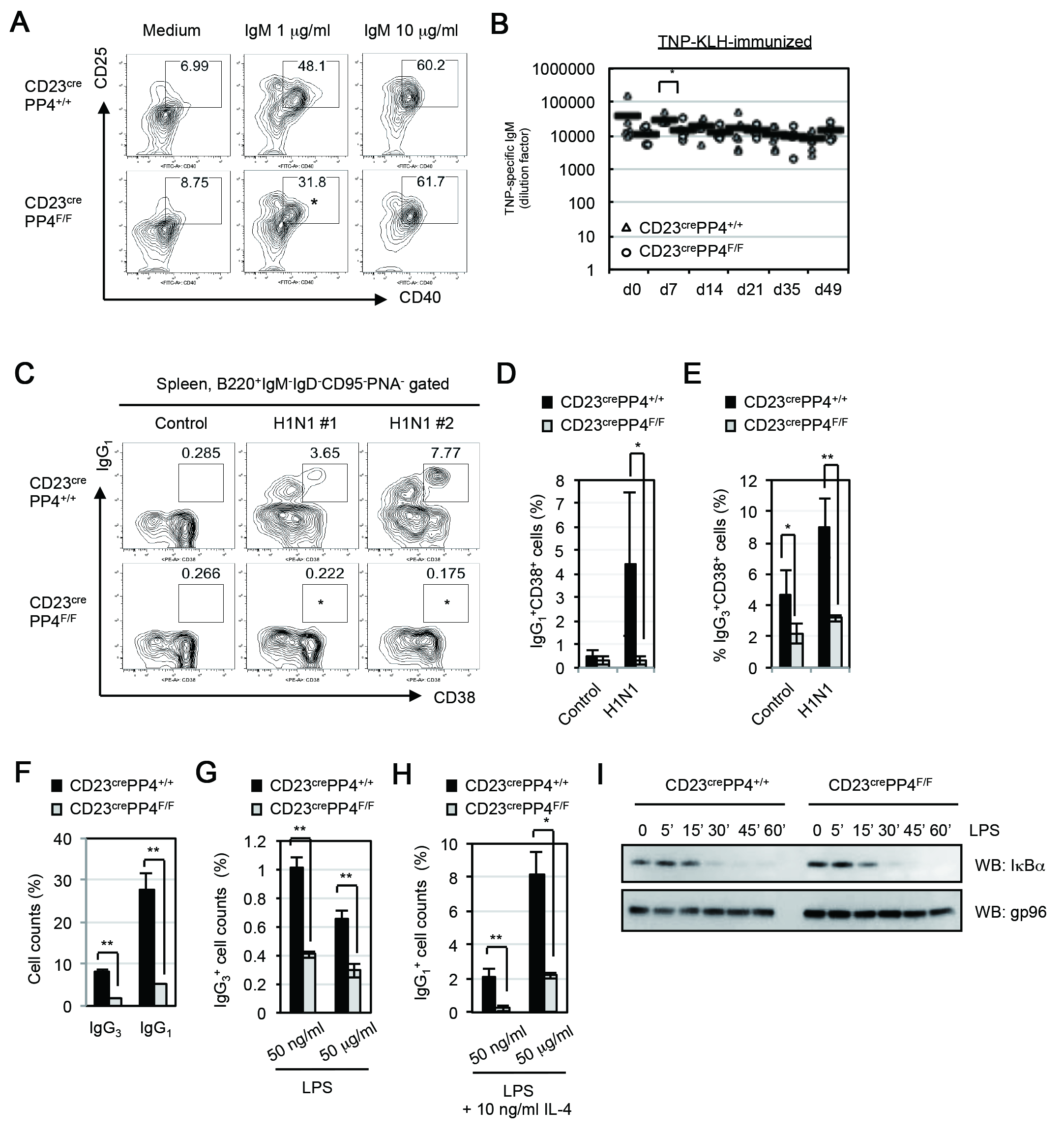

Supplement: Figure S3 — Various assays characterizing CD23crePP4F/F mice and their B cells. (A) FACS profile of CD40 vs CD25 expression by WT and CD23crePP4F/F B cells that were left unstimulated (Medium), or stimulated for 48 h in vitro in maintenance medium containing 1 µg/ml anti-IgM or 10 µg/ml anti-IgM. (B) Serum levels of TNP-specific IgM in WT and CD23crePP4F/F mice (n = 8–10/group) on the indicated days post-immunization with TNP-KLH. Data are values for individual mice and horizontal bars are geometric means. Results shown are from one experiment. (C) FACS profile of CD38 vs IgG1 expression by gated B220+IgM−IgD−CD95−PNA− splenic B cells from WT and CD23crePP4F/F mice at day 15 post-H1N1 infection. H1N1 #1 and #2 are identically infected mice in each group. Numbers in quadrants are the percentage of IgG1 +-switched B cells among total B cells. (D) Quantitation of the percentage of IgG1 +-switched B cells among total B cells from the data in (C). (E) Quantitation of the percentage of IgG3 +-switched B cells among B220+IgM−IgD−CD95−PNA−-gated B cells from the data in (C). For (C–E), results are representative of two independent experiments. (F) Quantitation of the percentage of IgG3 +- and IgG1 +-switched B cells (gated from B220+IgM−IgD− cells) among total B cells from the data in Figure 6A to 6D. (G) Quantitation of the percentage of IgG3 +-switched B cells (gated from B220+IgM−IgD− cells) among total B cells induced by various doses of LPS. (H) Quantitation of the percentage of IgG1 +-switched B cells (gated from B220+IgM−IgD− cells) among total B cells induced by various doses of LPS plus IL-4. (I) WB analysis of IκBα degradation in WT and CD23crePP4F/F B cells that were stimulated in vitro with 5 µg/ml LPS for the indicated times. gp96, loading control. Results are representative of two independent experiments. (TIFF) [file pone.0107505.s003.tiff]

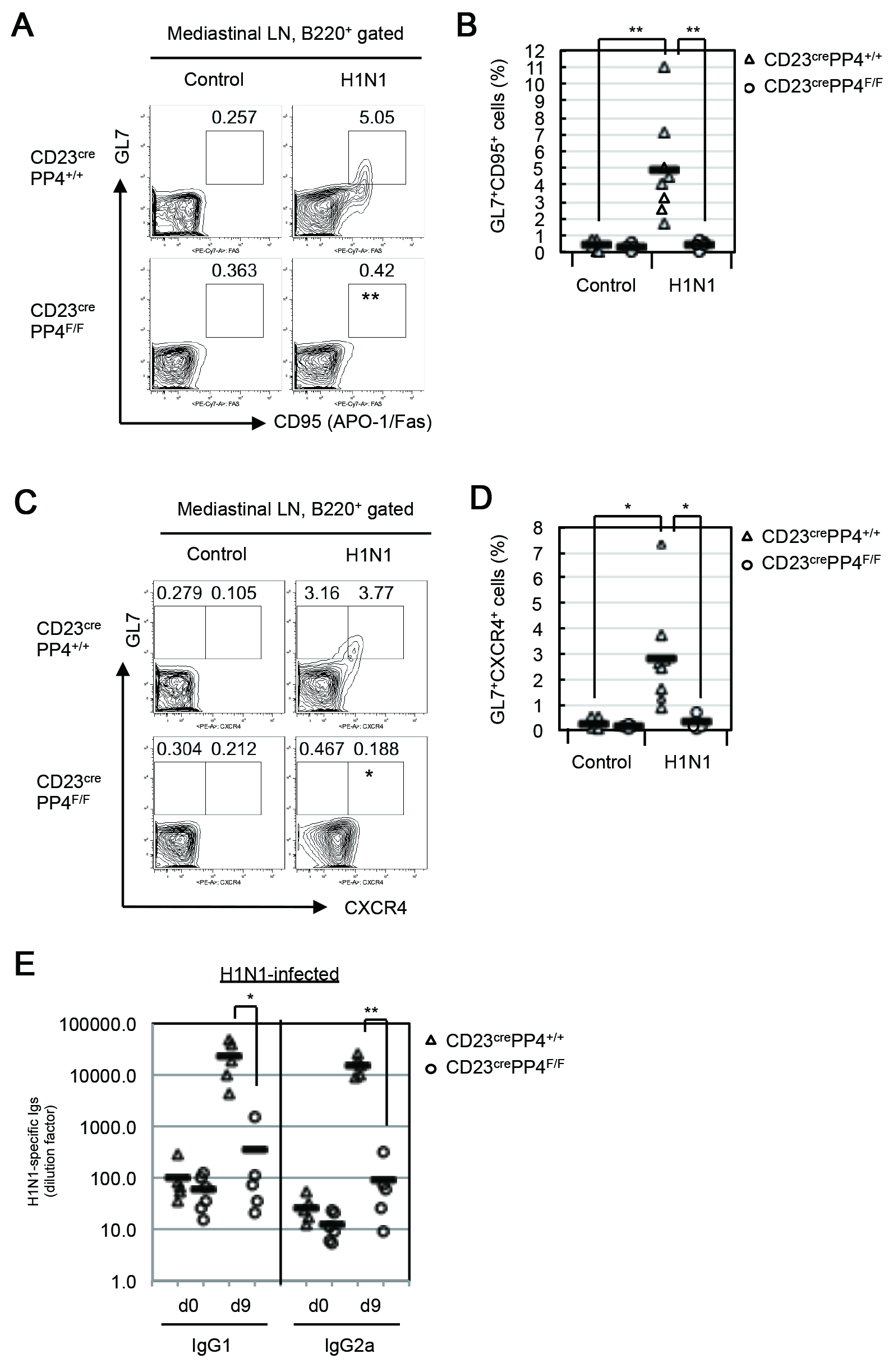

Supplement: Figure S4 — Impaired immune responses in CD23crePP4F/F mice infected with H1N1 virus. (A) FACS profiles of GL7 vs CD95 expression by B220+ lymphocytes isolated from the mediastinal lymph nodes in WT and CD23crePP4F/F mice (n = 4/group) at day 9 post-injection of PBS or H1N1 virus. (B) Quantitation of the percentage of GL7+CD95+ GC B cells among total B cells from the data in (A). (C) FACS profiles of GL7 vs CXCR4expression by B220+ lymphocytes isolated from the mediastinal lymph nodes in WT and CD23crePP4F/F mice (n = 4/group) at day 9 post-injection of PBS or H1N1 virus. (D) Quantitation of the percentage of GL7+CXCR4+ centroblasts among total B cells from the data in (C). For (A–D), results are representative of two independent experiments. (E) Quantitation of serum levels of H1N1-specific IgG1 and IgG2a in WT and CD23crePP4F/F mice (n = 5–6/group) before infection (d0) or at day 9 post-infection with H1N1. Data are from one experiment. (TIFF) [file pone.0107505.s004.tiff]

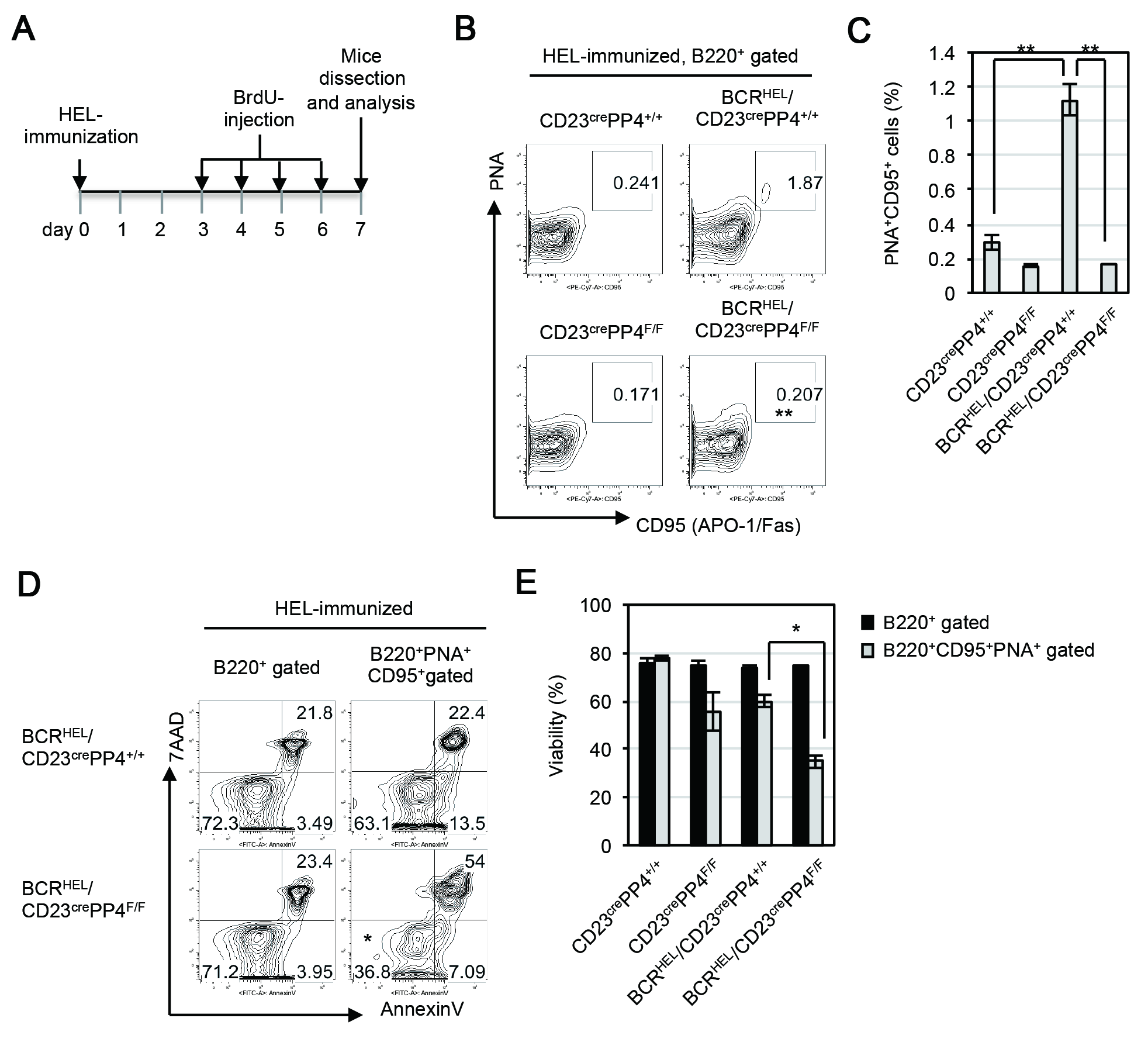

Supplement: Figure S5 — Reduced cell proliferation and reduced viability in transgenic mutant B cells from BCRHELCD23crePP4F/F mice with HEL immunization. (A) Illustration of the experiment procedure with HEL-immunization. BCRHELCD23crePP4+/+ and BCRHELCD23crePP4F/F mice (n = 4/group) were immunized with HEL in alum at day 0 and injected with BrdU from days 3 to 6. Mice were dissected at day 7 post-immunization and analyzed by FACS. (B) FACS profiles of PNA vs CD95 expression by B220+ splenocytes in BCRHELCD23crePP4+/+ and BCRHELCD23crePP4F/F mice at day 7 after immunization. (C) Quantitation of the percentage of PNA+CD95+ GC B cells among total splenic B cells from the data in (B). (D) FACS profiles of AnnexinV vs 7AAD expression by B220+ splenocytes in BCRHELCD23crePP4+/+ and BCRHELCD23crePP4F/F mice at day 7 after immunization. (E) Quantitation of the percentage of AnnexinV−7AAD− viable B cells among total B cells from the data in (D). (TIFF) [file pone.0107505.s005.tiff]
